# Factors Which Contribute to the Immunogenicity of Non-replicating Adenoviral Vectored Vaccines

**DOI:** 10.3389/fimmu.2020.00909

**Published:** 2020-05-19

**Authors:** Lynda Coughlan

**Affiliations:** Department of Microbiology, Icahn School of Medicine at Mount Sinai, New York, NY, United States

**Keywords:** adenovirus, adenoviral, vector, vaccine, immunogenicity

## Abstract

Adenoviral vectors are a safe and potently immunogenic vaccine delivery platform. Non-replicating Ad vectors possess several attributes which make them attractive vaccines for infectious disease, including their capacity for high titer growth, ease of manipulation, safety, and immunogenicity in clinical studies, as well as their compatibility with clinical manufacturing and thermo-stabilization procedures. In general, Ad vectors are immunogenic vaccines, which elicit robust transgene antigen-specific cellular (namely CD8^+^ T cells) and/or humoral immune responses. A large number of adenoviruses isolated from humans and non-human primates, which have low seroprevalence in humans, have been vectorized and tested as vaccines in animal models and humans. However, a distinct hierarchy of immunological potency has been identified between diverse Ad vectors, which unfortunately limits the potential use of many vectors which have otherwise desirable manufacturing characteristics. The precise mechanistic factors which underlie the profound disparities in immunogenicity are not clearly defined and are the subject of ongoing, detailed investigation. It has been suggested that a combination of factors contribute to the potent immunogenicity of particular Ad vectors, including the magnitude and duration of vaccine antigen expression following immunization. Furthermore, the excessive induction of Type I interferons by some Ad vectors has been suggested to impair transgene expression levels, dampening subsequent immune responses. Therefore, the induction of balanced, but not excessive stimulation of innate signaling is optimal. Entry factor binding or receptor usage of distinct Ad vectors can also affect their *in vivo* tropism following administration by different routes. The abundance and accessibility of innate immune cells and/or antigen-presenting cells at the site of injection contributes to early innate immune responses to Ad vaccination, affecting the outcome of the adaptive immune response. Although a significant amount of information exists regarding the tropism determinants of the common human adenovirus type-5 vector, very little is known about the receptor usage and tropism of rare species or non-human Ad vectors. Increased understanding of how different facets of the host response to Ad vectors contribute to their immunological potency will be essential for the development of optimized and customized Ad vaccine platforms for specific diseases.

## Introduction

### Use of Adenoviral Vectors as Vaccines for Infectious Disease

**Figure 1:**
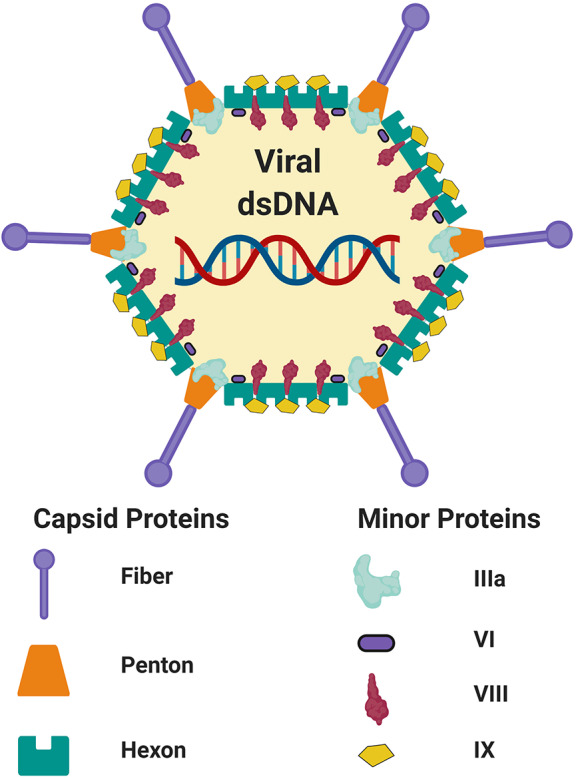
Schematic adenovirus structure. Schematic representation of the capsid and minor structural proteins of an adenovirus. Internal core proteins are not shown. The adenoviral virion contains linear double-stranded DNA genome (dsDNA). Figure is adapted from Russell (17) and was created with ©BioRender - biorender.com. Proteins are not to scale.

Adenoviruses (Ad) represent a promising vector platform for the development of vaccines for infectious disease, largely due to their safety and ability to stimulate robust cellular and/or humoral immune responses in multiple species (1–8), as compared with other genetic vaccine platforms (5, 9–12). Adenoviruses derived from humans and non-human primates (NHP) belong to the family *Adenoviridae* and the genus *Mastadenoviridae*, and are further subdivided into species A-G (i.e., for species A viruses, these are denoted HAdV-A followed by the type number). Accounting for the inclusion of many Ad recombinants (13, 14), ~103 human Ads (http://hadvwg.gmu.edu/) and >200 non-human Ad serotypes have been identified to date. Adenoviruses are non-enveloped viruses which contain a double-stranded DNA genome. The virion exterior is composed of three major structural proteins, the fiber, the penton base and the hexon [(15–18); Figure 1]. Recombinant Ad (rAd) vectors can easily be rendered replication-incompetent (non-replicating) through deletion of the essential viral gene E1 from their genome and can be vectorized for easy manipulation (16, 19–21). Further improvements to these first-generation Ad vectors have been developed in which the E3 region is also deleted, to accommodate a larger heterologous transgene capacity of ~7.5 kbp. Ad vectors display a number of desirable characteristics which make them particularly well-suited to prophylactic vaccine applications. Their genome is stable and easy to manipulate, they can be amplified and produced to high titers using various complementing cell lines which adhere with good clinical practice (GCP) procedures (22), and they have an outstanding track-record as safe and immunogenic vaccines in numerous human clinical trials (1–7, 23–25). Historically, the most commonly used rAd has been *Human mastadenovirus C*, Human Adenovirus Type-5 (HAdV-C5, *referred to Ad5 throughout this manuscript*). However, despite its well-characterized biology and robust immunogenicity, high seroprevalence has limited its widespread use in humans and has prompted the development and investigation of novel Ad species, either rare species human Ads (6, 20, 26) or those derived from NHPs (5, 8, 27), many of which have very low seroprevalence (27–31). For the purpose of this review, the nomenclature of Ad types will use reference to the vertebrate species from which the vector was derived (i.e., H for human or Ch for chimpanzee) followed by the virus type number, as outlined in Table 1. Human Ad vectors will include their assigned adenovirus species group (i.e., A-G). This nomenclature has been proposed to ICTV by Dr. Don Seto, George Mason University and Dr. James Chodosh, Harvard University (*personal communication*).

**Table 1:** Nomenclature of adenoviruses discussed in this review.

**Ad name (*this review*)**	**Description (vertebrate species, type)**	**Species/Group classification**	**Alternative names reported in the literature**
HAdV-B3	Human adenovirus type 3	B	Ad3, rAd3
^*^HAdV-B35	Human adenovirus type 35	B	Ad35, rAd35
HAdV-C2	Human adenovirus type 2	C	Ad2, rAd2
^*^HAdV-C5	Human adenovirus type 5	C	Ad5, rAd5, AdHu5
^*^HAdV-C6	Human adenovirus type 6	C	Ad6, rAd6, AdHu6
HAdV-D11	Human adenovirus type 11	D	Ad11, rAd11
^*^HAdV-D26	Human adenovirus type 26	D	Ad26, rAd26
HAdV-D28	Human adenovirus type 28	D	Ad28, rAd28
^*^HAdV-E4	Human adenovirus type 4	E	Ad4, rAd4
ChAdV-1	Chimpanzee adenovirus type 1	B2	ChAd1, AdC1, SAd21
^*^ChAdV-3	Chimpanzee adenovirus type 3	C	ChAd3
ChAdV-7	Chimpanzee adenovirus type 7	E	AdC7, ChAd7, SAd24, Pan7
^*^ChAdV-63	Chimpanzee adenovirus type 63	E	ChAd63
ChAdV-68	Chimpanzee adenovirus type 68	E	AdC68, ChAd68, SAd25, Pan9, ChAdOx2
^*^ChAdOx1	Chimpanzee adenovirus type Y25	E	Y25
SAdV-11	Simian adenovirus type 11	Yet undefined	SAd11, sAd11
SAdV-16	Simian adenovirus type 16	Yet undefined	SAd16, sAd16
SAdV-23	Simian adenovirus type 23	E	ChAdV-6, AdC6, ChAd6, Pan6
^*^PanAdV-3	Pan (*paniscus*) adenovirus type 3	C	PanAd3

*Adenovirus classification for human and non-human Ad vectors referred to in this review. The nomenclature of Ad vectors derived from non-human primates, including chimpanzees, is not standardized, resulting in the confusing use of multiple names assigned by individuals who vectorized these constructs. In this review text, we propose to follow current standards for human Ad vectors, such as HAdV-C5, as outlined by ICTV, for descriptions of Ad vectors derived from chimpanzees or non-human primates. Abbreviated “alternative” names are used in Table 2 due to space constraints. H, human; Ch, chimpanzee; S, simian*.

* *Vectors used in human clinical trial*.

The use of non-replicating viral vectors as a vaccine platform has several advantages over other vaccine formulations (i.e., recombinant protein, inactivated particles). Viral vectored vaccines retain some characteristics of a live attenuated vaccine in terms of their ability to enter target cells, engage intracellular trafficking pathways to deliver their genome and facilitate antigen (Ag) expression and subsequent Ag-presentation *in vivo*, but possess additional safety features. Furthermore, in order to drive the expression of substantial quantities of transcripts which correspond to the encoded vaccine Ag, non-replicating Ad vectors make use of powerful exogenous promoters, such as the cytomegalovirus (CMV) promoter (32). Unlike recombinant protein or inactivated vaccines in which antigen quantity is limited to the input vaccine dose, the use of exogenous promoters facilitates more sustained transgene antigen expression *in vivo*.

In general, Ad vectors are well-established to stimulate CD8^+^ T cell responses directed toward transgene Ag, with selected Ad types confirmed to elicit robust cellular immunity in both animal models (8, 32–36) and humans [(1, 3, 6, 23, 33, 37); see Table 2]. Memory CD8^+^ T cell responses elicited following vaccination with Ad vectors exhibit an extended contraction phase (38). Importantly, the persistent Ag expression following immunization with Ad vaccines enables the induction of sustained immune responses (5, 36, 39–41), making them very attractive vaccine vectors for conferring long-lasting immunity. It is believed that the prolonged expression of vaccine Ag facilitates the maintenance of effector CD8^+^ T cells while simultaneously permitting their differentiation into central memory populations (36). Improved understanding into how Ad vectors prime and maintain such long-lived responses will be crucial not only in designing improved Ad vaccines, but also other vaccine platforms which are optimized for diverse disease targets. However, the precise factors which contribute to the robust immunogenicity associated with particular Ad-vectored vaccines are currently unclear.

**Table 2:** Summary of the comparative immunogenicity of diverse AdV vaccines.

**A. Ad vectors compared**	**B. Vaccine antigen**	**C. Parameters used to define hierarchy of immunogenicity**	**Optimal vector**	**PMID**
**SPECIES: MICE**				
**Human Ads** **B:** Ad34, Ad35 **C:** Ad5, Ad6 **D:** Ad24 **Non-human Ads** **B:** ChAd30 **C:** ChAd3, PanAd3, PanAd1, PanAd2, ChAd11, ChAd19, ChAd20, ChAd24, ChAd31 (listed in order of immunogenicity) **E:** ChAd63, ChAd83, ChAd6, ChAd9, ChAd10, ChAd43, ChAd55, ChAd147, ChAd4, ChAd5, ChAd7, ChAd16, ChAd38, ChAd146, ChAd149, ChAd150 (listed in order of immunogenicity)	HIV-1 *gag*	**Maintenance of Gag^+^CD8^+^IFNγ^+^ responses with dose de-escalation (10^10^-10^6^ vp):** Ad5, Ad6 > Ad24 > Ad35 > Ad34 (*Balb/c mice*, intramuscular immunization) • Ad5 and Ad6 were capable of eliciting T cell responses at a vaccine dose of 10^6^ viral particles (vp). Ad24 = 10^8^ vp, Ad35 = 10^9^ vp, and Ad34 = 10^10^ vp. **Maintenance of Gag^+^CD8^+^IFNγ^+^ responses with dose de-escalation (10^10^-10^6^ vp):** ChAd3, PanAd3 > ChAd63 > PanAd1 > as listed in column A (*Balb/c mice*, intramuscular immunization) • **26** chimpanzee adenoviral isolates were screened for immunological potency. • Group C Ad vectors were most potently immunogenic, followed by Group E. Group B ChAd30 was weakly immunogenic. • ChAd3 and PanAd3 were capable of eliciting T cell responses at a dose of 10^6^ viral particles (vp), ranking them as comparable with the immunogenicity of Ad5. • ChAd63 also elicited T cell responses at 3 × 10^6^ vp making it only slightly less immunogenic than ChAd3 and PanAd3.	**Ad5, Ad6** **ChAd3, PanAd3, ChAd63**	22218691
**Human Ads** **B:** Ad35 **C:** Ad5 **D:** Ad28 **Non-human Ads** **C:** ChAd3 **E:** ChAd63 **ND:** SAd11, SAd16	SIV *gag*	**Magnitude and protective efficacy of CD8^+^ with dose de-escalation (10^9^-10^7^ particle units PU):** Efficacy ranking: Ad5+ChAd3 > Ad28+SAd11 > ChAd63 > SAd16 > Ad35 (*C57BL/6 mice*, sub-cutaneous immunization) • Study performed a dose titration of vaccines with detailed phenotyping of T cell response at peak and memory timepoints. • Ad5, Ad28, SAd11, and ChAd3 were comparable in conferring CD8^+^ mediated protection in challenge model using gag-expressing *Listeria monocytogenes*.	**Ad5, Ad28** **ChAd3** **SAd11**	23390298
**Human Ads** **B:** Ad35 **C:** Ad5 **D:** Ad28 **Non-human Ads** **C:** ChAd3 **E:** ChAd63 **ND:** SAd11, SAd16	SIV *gag* *eGFP*	**Memory (D70) Gag Tetramer^+^CD8^+^ at 10^8^ PU and protection from challenge:** Ad5, ChAd3 > ChAd63 (*C57BL/6 mice*, sub-cutaneous immunization) • The protective efficacy of Ad vectors in a murine challenge model of *gag*-expressing *Listeria monocytogenes* was tested. • Ad5 and ChAd3 were most potently protective, followed by ChAd63. • Vaccination with Ads expressing reporter eGFP resulted in higher frequencies of eGFP^+^ CD11c^+^ dendritic cells in draining lymph nodes (dLNs) for Ad5, ChAd3 > ChAd63. • Transcriptional profiling of dLNs revealed that the most potently protective vectors, Ad5 and ChAd3, exhibited weak induction of IFN-stimulated genes, unlike other Ad vectors (8–24 h). • More pronounced upregulation of IFN-responsive gene modules in dLNs at 24 h were associated with reducing Ad transgene expression levels and thereby limiting their immunological potency.	**Ad5** **ChAd3, ChAd63**	25642773
**Human Ads** **B:** Ad11, Ad35, Ad50 **C:** Ad5 **D:** Ad26, Ad48, Ad49	SIV *gag*	**Maintenance of Gag^+^CD8^+^IFNγ^+^ responses with dose de-escalation (10^10^-10^7^ vp):** Ad5 > Ad26 (*C57BL/6 mice*, intramuscular immunization) • A head-to-head comparison of species B and D Ad-based vaccine vectors was performed compared with species C Ad5. • At a dose of 10^7^ vp, Ad5 and Ad26 were more potent than all other Ad vectors, but only Ad5 was immunogenic at a dose of 10^6^ vp.	**Ad5, Ad26**	17329340
**Human Ads** **C:** Ad5 **D:** Ad26 **Non-human Ads** **E:** AdC6 (SAdV-23), AdC7 (SAdV-24)	HIV-1 *gag* Rabies *glycoprotein*	**Magnitude of Gag^+^CD8^+^IFNγ^+^ responses at 10^9^ or 10^8^ vp:** Ad5 > Ad26, AdC6, AdC7 (*Balb/c mice*, intramuscular immunization) • At 10^10^ vp gag-specific CD8+ responses were comparable for Ad5, Ad26, AdC6, and AdC7. • At 10^8^ vp, Ad5 was superior to all. **Induction of NAbs to Rabies GP and Protective Efficacy at 10^11^-10^9^ vp:** Ad5 > Ad26, AdC6, AdC7 (*ICR outbred mice*, intramuscular immunization) • At all doses neutralizing antibodies considered protective according to comparison with WHO standard serum samples (>0.5 IU) were induced, but Ad5 was the best. • Ad5 had superior protective efficacy from challenge with rabies virus strain CVS-24 at all doses, survival with other Ads was reduced, even at 10^11^ vp. • Ad26 displayed 60% survival, AdC6 was ~50% survival at 10^11^ vp.	**Ad5, Ad26** **Ad26, AdC6**	20686035

*We are only including murine studies that performed a head-to-head comparison of vector immunogenicity for Ad vaccines derived from more than three Ad species groups. Please note, vector type names are listed as shortened versions due to space constraints within the Table. IFNγ, interferon gamma; vp, viral particle; PU, particle unit; HIV, human immunodeficiency virus; SIV, simian immunodeficiency virus; eGFP, enhanced green fluorescent protein; dLN, draining lymph node; GP, glycoprotein. Abbreviated “alternative” names for Ad vectors are used in this table due to space constraints. See Table 1 for their standardized nomenclature*.

It is widely appreciated in the Ad vaccine field that Ad vectors can act as a “self-adjuvant,” allowing the stimulation of multiple innate immune signaling pathways upon viral entry, which can augment the immunogenicity of the encoded Ag (although conversely, stimulation of certain signaling pathways can also be detrimental to their immunogenicity, as discussed below). Although we have some understanding of how individual pathways work *in vitro* in defined cell types (i.e., dendritic cells, macrophages), understanding how these pathways intersect, or cooperate in the development of protective immunity *in vivo*, is complex and our understanding is incomplete. Additionally, it is apparent that there is a clear hierarchy of immunological potency when evaluating distinct Ad species and serotypes as vaccine vectors in animal models. Although a few selected vectors display robust immunogenicity *in vivo* which is comparable to that of Ad5, most are less immunogenic (5, 8, 15, 29, 31), and there are considerable differences in the phenotype and functionality of immune response elicited (8, 42, 43). In recent years, investigators have begun to identify several crucial factors which could contribute to these profound disparities in immunological potency. It is now believed that differences in (i) cellular receptor and/or co-receptor usage, viral entry, trafficking, endosomal escape, and *in vivo* tropism can contribute to the (ii) differential activation of innate immune signaling which influences subsequent immune responses (44). In addition, it is apparent that the (iii) magnitude and persistence of transgene expression can also shape the ensuing immune response (33) and all of these factors are in turn affected by the (iv) vaccine dose (8) and route of administration (45). Increased understanding of, and implementation of efforts to overcome these striking differences in immunological potency or quality, will absolutely be required for the development of optimal Ad vectors for clinical use.

#### Viral Entry and Cellular Tropism

##### Entry in non-immune cells

**Figure 2:**
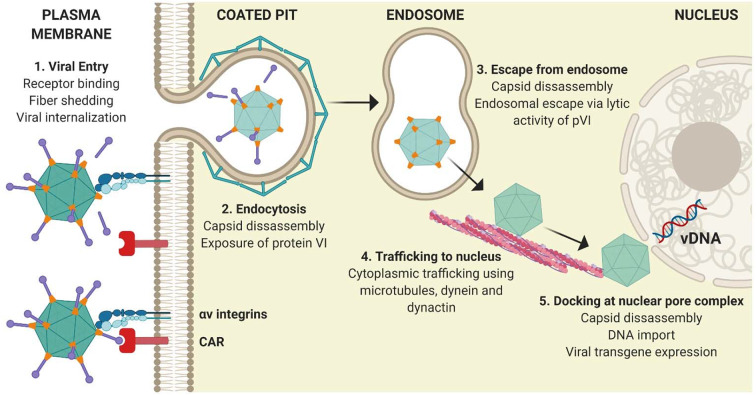
Classical adenoviral entry pathway. **(1)** Attachment of the Ad5 adenoviral fiber knob mediates primary receptor interactions with CAR, followed by an interaction between the RGD motif within the penton base and cellular integrins on the surface of cells, which initiate partial capsid disassembly by fiber shedding. **(2)** Internalization of virions is mediated by integrins. **(3)** Exposure of pVI from the virion interior facilitates its lytic activity and allows endosomal escape. **(4)** Partially disassembled nucleocapsid cores traffic to the nucleus using the microtubule network before docking at the nuclear pore complex. **(5)** Viral DNA enters the nucleus and viral transgene expression is initiated. Figure is updated from Coughlan et al. (18) and was created with ©BioRender - biorender.com.

As a result of extensive study over the past few decades, we have a clear understanding of the *in vitro* tropism determinants of vectors derived from species C HAdVs (i.e., HAdV-C5/Ad5) (18). The classical entry pathway of rAd5-based vectors in non-immune cells is mediated by binding of the fiber knob domain (Figure 2) to the Coxsackie and Adenovirus receptor (CAR). Following this “docking” interaction, viral internalization is facilitated through interactions between the arginine-glycine-aspartate (RGD) motif within the viral penton base and cellular integrins (namely αvβ3 and αvβ5) on the surface of cells (46, 47). Adenovirus capsid disassembly then proceeds in a systematic and stepwise process. Initial binding to CAR is a motile interaction, whereas the subsequent interaction with immobile αv integrins results in the ripping or shedding of fibers from the virion, initiating partial disassembly of the virion at the plasma membrane (48, 49). It was previously believed that endosomal escape was pH-dependent (50). However, it has subsequently been demonstrated that exposure of protein VI from the capsid interior (51) at the cell surface, as a result of mechanical strain induced by the antagonistic CAR:integrin interaction (48), facilitates access to the cytoplasm through the action of its pH-independent membrane lytic activity (52–54). Following endosomal escape, the virion is transported to the nuclear pore complex via the microtubule network (50, 52). Once the virion has docked at the nuclear pore complex, interactions with cellular proteins trigger further capsid disassembly and allow the viral DNA to extrude into the nucleus for subsequent gene expression (18).

However, unlike Ad5, adenoviral types derived from species B or D viruses such as HAdV-B35, HAdV-B3, HAdV-D11, or HAdV-D26 can use alternative binding/entry factors or receptors to CAR, such as CD46 (55–57), desmoglein-2 (DSG-2) (58), or sialic acid (59). The post-entry steps of these rare species Ad viruses in diverse cell types are not as well-characterized as the CAR-mediated entry of Ad5. However, it is considered that the use of alternative entry pathways or different receptors can not only result in differences in endosomal escape, trafficking to the nucleus and subsequent transgene expression (60), but can also impact on the *in vivo* tropism of the vector following different routes of vaccine administration (i.e., intramuscular vs. intranasal). As a result, triggering of innate immune signaling pathways may also differ at each step of the entry process (33, 61). Less efficient trafficking pathways could result in weak or limited induction of cytokines/chemokines (62), or increased uptake in cell types which result in vector degradation with minimal transgene expression (63–66). Consequently, such differences between diverse Ad vectors can impact on the magnitude and phenotype of the ensuing adaptive immune response when they are used as a vaccine platform (8, 33, 44).

##### Entry in immune cells

*Macrophages*. In addition to the classical *in vitro* entry pathways described above, Ad vectors can infect mononuclear phagocytes efficiently both *in vitro* and *in vivo*, independently of their described surface receptors (i.e., CAR, DSG-2) (67), which are absent on murine macrophages. *In vivo* interactions with tissue resident macrophages, such as Kupffer cells in the liver or alveolar macrophages in the lung, can result in scavenging and degradation of significant amounts of input Ad vector (64–66). Not only can these interactions result in limited transgene expression which could affect the therapeutic efficacy, but the phagocytosis of Ad particles can trigger inflammatory responses (68, 69), leading to undesirable off-target toxicity (70). This is a particularly important consideration for therapeutic applications which require systemic administration or are designed for use in immunocompromised individuals (i.e., oncolytic viral therapy for disseminated metastases) (18). As a result, efforts have been made to characterize the mechanisms of Ad viral entry in macrophages and to better understand how these interactions contribute to the induction of inflammatory responses within defined anatomical compartments following different routes of administration (i.e., intramuscular, intranasal vs. intravenous).

Opsonization of Ad viral particles by complement or antibodies (natural or anti-viral) can bridge entry into macrophages by engaging Fc-receptors (FcRs) or complement receptors (71–73). Additionally, a role for scavenging receptors in facilitating viral entry into murine macrophages, both *in vitro* and *in vivo*, has been outlined (62, 67, 74). Scavenging receptors are a heterogenous and structurally diverse family of receptors capable of interacting with endogenous proteins and lipids, microbial ligands, and non-opsonized particles, including viruses (75). In addition to contributing to the clearance of particulate Ag, scavenging receptors have been implicated in innate immune sensing, due to their ability to recognize pathogen-associated molecular patterns (PAMPs). Murine SR-A1 (74), SR-AII (76), and MARCO (SR-A6) (67) have been described as receptors for rAd vectors, and MARCO^+^ marginal zone macrophages in the spleen have been shown to accumulate Ad5-based vectors following intravenous (*i.v*.) delivery in mice (28, 77). The fiber knob (i.e., SR-A1), or hexon protein has been implicated in mediating these interactions (i.e., SR-AII and SR-A6). Interestingly, SR-A6 (MARCO) was shown to not only facilitate entry and efficient gene transduction with Ad5, but also with HAdV-C2, HAdV-B35, and HAdV-D26 (67). The mechanism of interaction between Ad and SR-AII/SR-A6 was proposed to involve the negative charge conferred by specific hypervariable regions (HVRs) of the viral hexon, namely HVR1. In support of this, preferential scavenging of negatively charged particles has previously been shown to contribute to the differential recognition of Ad vectors by macrophages *in vivo* (76, 78, 79).

**Figure 3:**
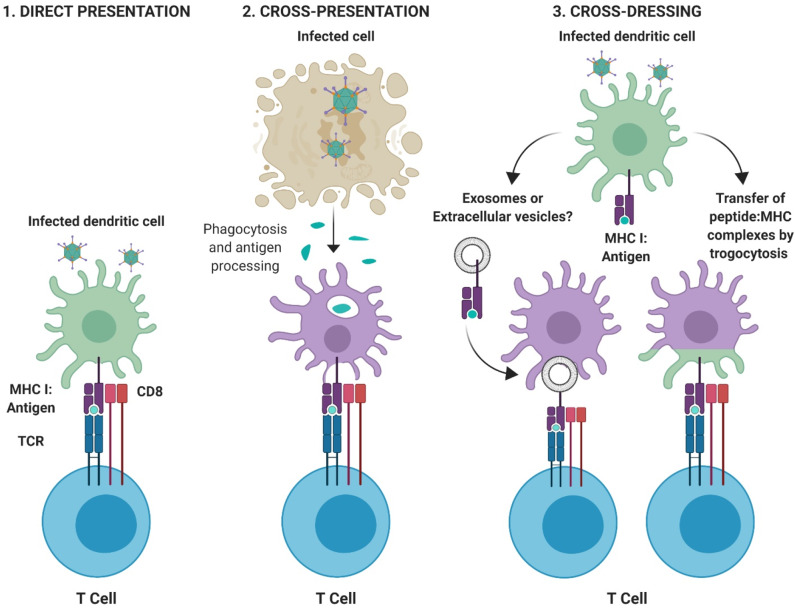
Pathways for antigen presentation. **(1)** *Direct-presentation:* Antigen from virally infected cells is degraded by the proteasome and processed for peptide presentation to CD8^+^ T cells on MHC I. **(2)** *Cross-presentation:* Antigen fragments derived from virally infected cells are phagocytosed by professional APCs and peptide processed and presented to T cells via appropriate MHC molecules. **(3)** *Cross-dressing:* Peptide:MHC complexes can be acquired through transfer via extracellular vesicles or exosomes, or by a process of membrane gnawing called trogocytosis. Figure is adapted from Yewdell and Dolan (85) and was created with ©BioRender - biorender.com.

*Dendritic cells*. Dendritic cells (DCs) are a specialized subset of professional Ag presenting cells which are central to the development of protective immunity. DCs can process Ag using several methods; (i) direct presentation, in which an infected DC presents peptide:major histocompatibility complex class (MHC) complexes directly to T cells, (ii) cross-presentation in which Ag derived from other infected cells is phagocytosed by DCs, processed and then presented to T cells, and (iii) cross-dressing (80), in which peptide:MHC complexes are acquired from other professional or non-professional antigen presenting cells (APCs) and are transferred to the DC through a process of trogocytosis, in which fragments of the plasma membrane containing the MHC complex merges with the recipient cell. Cross-dressing is also hypothesized to occur through the intercellular transfer of pre-formed peptide:MHC complexes by extracellular vesicles, such as exosomes [(81–84); Figure 3].

A critical role for DCs in the robust induction of CD8^+^ T cell responses following immunization with rAd vectors has been demonstrated (86, 87). It has been shown that CD8^+^, but not CD4^+^ T cell responses elicited by Ad vaccines, are dependent on cross-presentation by specific sub-populations of DCs, including CD8α^+^ DCs (33). In support of this, Ag-specific CD8^+^ T cell responses in BATF3-deficient mice (*Batf3*^−/−^), which preclude the development of the latter DC population (CD8α^+^ DCs), were shown to be ~80% lower following immunization with several Ad vectors (33). DCs infected with Ads upregulate MHC, as well as co-stimulatory molecules including CD40, CD80, or CD86, leading to the activation or maturation of DCs (88), in a manner which is believed to be dependent on nuclear factor kappa-light-chain-enhancer of activated B cells (NF-κB) signaling (89). The maturation of murine DCs has been proposed to be mediated by the fiber knob domain of Ad5 (90). *In vivo* experiments in mice have shown that uptake of rAd5-based vaccines in draining lymph nodes (dLNs) following intramuscular (*i.m*.) or subcutaneous (*s.c*.) vaccination is highest in CD11c^+^ CD8^−^ B220^−^ DCs, although CD11c^+^ CD8^+^ B220^−^ DCs were the most potent for eliciting naïve Ag-specific T cell proliferation (86). As previous studies have shown that targeting vaccine Ag to defined populations of DCs can improve immunity and vaccine efficacy (91, 92), efforts are ongoing to better understand the precise interactions between diverse Ad types and DC subpopulations *in vivo*, as well as how we can engineer Ad vectors which are targeted to specific receptors on the surface of DCs (93, 94).

Receptors on the surface of dendritic cells (DCs) can permit entry of Ads independently of the classical CAR receptor, which is largely absent on DCs (95). Receptors proposed to be involved in Ad viral entry into human or murine DCs include Dendritic Cell-Specific Intercellular adhesion molecule-3-Grabbing Non-integrin (DC-SIGN) (95), CD46 (61, 96, 97), or CD80/CD86 (98). A vector based on chimpanzee Ad vector 1, ChAdV-1, was shown to efficiently transduce CD46-expressing murine DCs *in vitro* (97). Recombinant Ad5 vectors pseudotyped with the fiber knob from Ad3 (99) or porcine adenovirus type 4 (100), can increase entry into human DCs, via CD80/CD86 and surface glycans, respectively. Similarly, pseudotyping rAd5 with the fibers from species B, HAdV-B16 or species D, HAdV-D37 displayed increased entry into murine DCs compared with unmodified rAd5 (101). Interestingly, in the latter study, increased entry into DCs was not associated with improved cellular immunity following subcutaneous immunization. In support of this, species B Ad vectors, including Ad35, were long considered to hold great potential as potently immunogenic vaccine vectors due to their increased ability to target both myeloid and plasmacytoid human DCs via CD46. However, these vectors were subsequently shown to be some of the least potent Ad vaccines *in vivo* (5, 8). These observations and findings are important in highlighting that multiple parameters, such as post-entry intracellular trafficking kinetics or differential activation of innate immune signaling pathways, not just viral tropism, likely play key roles in the induction of robust immunogenicity.

#### Innate Immune Responses to Ad Vectors

The ability of diverse rAd vaccines to elicit robust cellular immune responses and confer protective immunity in animal models makes them an attractive vector platform for vaccine development (Table 2). In addition to this, their safety in clinical applications, compatibility with clinical-grade manufacturing and scale-up (102, 103) and their suitability for long-term storage (104), or thermo-stabilization (105–107) and stockpiling for cold-chain free storage, has solidified their appeal as vaccines for major infectious diseases (108). It is this clear translational potential which has emphasized the importance of improving our understanding of the mechanisms which underlie differences in the immunological potency of diverse Ad types. Ad vectors are capable of triggering multiple innate immune sensors at several steps in the viral entry pathway (109, 110), in a process which does not require viral replication or gene expression (111). Viral penton RGD:cellular integrin-mediated internalization and subsequent escape from the endosome is considered to be a crucial step in activating many innate immune responses to Ad vaccines (112). It is considered that preferential stimulation (or avoidance) of defined innate immune signaling pathways could impact on the downstream immunogenicity of distinct Ad vectors. With regard to assessing the differential stimulation of innate immune signaling pathways, this is complicated by the fact that many vectors have not been compared side-by-side, and published data proposing roles for these pathways in the immunological potency of different Ad vectors are often contradictory. However, a number of pathways which have been implicated in innate immune sensing of Ad vectors and the caveats associated, are described below.

##### TLRs

Recombinant Ad vectors contain PAMPs which can be sensed by cell-surface or endosomal pattern recognition receptors (PRRs) such as the Toll-like receptors (TLRs). TLRs implicated in the sensing of Ad vectors include TLR2 (113), TLR4 (114), and endosomally located TLR9 (61, 113), which can trigger the down-stream activation and transcription of anti-viral genes including NF-κB, mitogen-activated protein kinases (MAPK) and interferon-regulatory factors (IRFs). The intracellular adaptor protein MyD88 has been reported to play a major role in the induction of Ag-specific cellular immune responses following TLR-mediated sensing of Ad vaccines (109, 113). Importantly, the ability to engage multiple MyD88-dependent signaling pathways simultaneously, is believed to contribute to the robust immunogenicity associated with Ad vaccines. However, their immunological potency is also attributed to the fact that innate immune activation by recombinant Ad vaccines can occur not only via TLR-dependent mechanisms, but also through numerous TLR-independent pathways (86, 109, 110, 113, 114).

##### cGAS/STING

The viral DNA itself can play a crucial role in triggering innate immune responses. In recent years it has been demonstrated that following rupture of the endosomal membrane, Ad viral DNA can also be sensed by the cytosolic DNA sensor cGAS (115, 116). The engagement of cGAS triggers a signaling cascade involving the adaptor STING (117) and activation of the kinase TBK1, which initiate the induction of IRF3-responsive genes (115), such as Type I interferons (IFNs). It has been shown that the absence of cGAS or STING results in reduced activation of early innate immunity (i.e., IFN-β, cytokines, chemokines) but does not impact adaptive anti-vector immune responses in mice. However, the latter studies were performed in the context of *i.v*. delivery and anti-vector, not transgene-specific immunity (116), and as such, the relative importance of DNA sensor pathways in the immunogenicity of Ads as vaccine vectors is less clear. It has recently has been suggested that Ag expression is a more crucial predictor of Ag-specific memory T cells (33), as abrogation of STING and Type I IFN responses during Ad vaccination in mice merely altered the early kinetics of CD8^+^ T cells, but did not impair the magnitude of T cell memory responses (33). In the latter study, it was shown that STING could act as a dominant innate PRR sensor for many Ad vectors. Interestingly, abrogation of STING accelerated the kinetics of Ag-specific T cell responses following vaccination with ChAdV-63, a chimpanzee Ad vector, but was dispensable for the early induction of CD8^+^ T cell responses for Ad5 and rare species HAdV-D28-based vaccines (33). This supports the idea that a complex interplay between multiple PRR-mediated signaling pathways exists, and that different Ad vectors are differentially impacted by these pathways. Our understanding of this is confounded by differences in receptor usage, *in vivo* tropism, engagement of PRRs in diverse hematopoietic and non-hematopoietic cells, and by differences in putative PAMPs on diverse Ad particles.

##### The NLRP3 inflammasome

In addition to inducing the expression of anti-viral genes, infection with Ad vectors also triggers pro-inflammatory responses through cytosolic DNA-sensing mechanisms which are independent of TLR9 and IRFs (111). In macrophages, recognition of Ad viral DNA has been shown to be mediated by the innate cytosolic molecular complex, or inflammasome, in a process involving NLRP3 and (ASC), which is independent of viral gene expression or replication (117). The multi-protein inflammasome complex mediates caspase-1 activity, resulting in the processing of pro-interleukin-1β into its active and secreted form. IL-1β subsequently induces signaling cascades of pro-inflammatory cytokines and chemokines through the IL-1RI both *in vitro* and *in vivo* in response to Ad infection. However, alternative and contradictory mechanisms of immune activation in macrophages by Ads have also been identified which are independent of the NLRP3 inflammasome and its components. Di Paolo et al. showed that direct interactions between the RGD motif within the penton base of the Ad virion and the β3 subunit of integrins on the surface of macrophages were responsible for activating IL-1α. The authors also proposed that IL-1α, not IL-1β, was the predominant activator of innate immune responses to Ad5 *in vivo* (77).

One very important caveat which complicates our ability to systematically investigate how innate immune responses contribute to downstream adaptive immunity to Ad vaccines, is that many studies are performed *in vitro*, using defined non-immune epithelial/endothelial cells or cultured immune cells including macrophages or dendritic cells (110, 115, 118). These cell type-specific findings often contradict subsequent *in vivo* studies using transgenic mice in which these “critical” mediators of innate immunity are knocked-out (77, 116). For example, despite numerous reports describing an important role for TLR9 *in vitro*, comparisons of wildtype and TLR9^−/−^ mice have demonstrated that the impact of TLR9 in innate immune sensing of Ad particles *in vivo* is minimal (77), at least for *i.v*. administration of Ad5. Similarly, although cGAS or STING were shown to be pivotal in *early* immune sensing of Ad *in vivo*, studies using cGAS^−/−^ or STING^−/−^ mice showed that these molecular effectors have little impact on subsequent adaptive immunity and antibody production (116). These discrepancies are obviously further complicated by the known capacity of Ad vectors to engage multiple innate signaling pathways simultaneously, rendering individual pathways at least partially redundant *in vivo* (110). In addition to this, differences in the route of Ad vector delivery (i.e., *i.m, i.v.*, or intranasal) and access to different cell types, the multiplicity of infection (MOI) or injected dose, the timing or method of analysis reported in published work and the use of non-human or rare species Ad vectors with differential receptor usage, also limits our ability to fully dissect out the key contributing pathways (118, 119). Collectively, these factors highlight the many challenges facing the field and explain why we currently lack consensus on precisely which innate signaling pathways could contribute to protective immunity following vaccination with diverse Ad vectors.

##### Stimulation of type I IFNs

It has been proposed that *minimal* induction of Type I IFNs (44, 102, 120), in conjunction with sustained transgene expression (33), are hallmarks of potently immunogenic Ad vaccine vectors *in vivo*. Excessive stimulation of Type I IFN pathways at early time-points following immunization has been shown to lead to decreased transgene expression and subsequently reduced Ag-specific antibody (Ab) responses, following immunization with a chimpanzee Ad vector, ChAdV-68 (120). The authors demonstrated that these effects could be reversed by immunizing mice which have a defective type I IFN receptor IFNAR^−/−^, resulting in an increased Ab response, thereby confirming that Type I IFN stimulation can have a detrimental impact on humoral immunity directed toward the Ad-encoded transgene Ag (120). In support of these findings, Quinn et al. also showed that abrogation of Type I IFN and STING could increase transgene expression from rAd vaccines, and that the development of protective cellular immunity correlated with this increased transgene expression (33). Following immunization with rare species and non-human Ad vectors, the authors used a systems biology-based, gene expression analysis approach at several time-points to confirm the differential modulation of IFN responsive genes. They determined that Type I and Type II IFNs were upregulated at 8 h post-immunization, which was followed by the induction of ISGs by 24 h. Interestingly, the most protective rAds identified in their study (i.e., Ad5 and chimpanzee Ad, ChAdV-3) exhibited the weakest transcriptional activation of these pathways. However, only the impact of innate gene activation on CD8^+^ T cell responses, but not transgene-specific Abs, was investigated (33). Nonetheless, collectively, these studies support the concept of robust, persistent Ag expression combined with low innate gene stimulation in contributing to the potency of rAd vaccines (33, 102). This is also supported by the knowledge that relative to rare species human and non-human Ad vectors (33, 120), immunization with the potently immunogenic Ad5 vector is well-established to result in robust, persistent Ag expression (33, 36, 41, 121, 122), while triggering minimal Type I IFN responses *in vivo*. Future studies which aim to comprehensively characterize the contribution of early innate immune activation and correlate this with the downstream immunological potency and efficacy of lead Ad vaccine platforms will be required.

#### Magnitude and Persistence of Antigen Expression

Non-replicating Ad5-based vectors are well-established for their ability to confer robust transgene expression following immunization (36). Furthermore, low level transgene expression can persist long-term (41, 122), with transcriptionally active Ad vector genomes being maintained in muscle at the injection site, or within draining lymph nodes (36, 122), depending on the route of administration. As previously outlined, it has been proposed that it is this magnitude and persistence of transgene Ag expression which is crucial for the induction of robust and protective T cell responses following Ad vaccination (33). As described above, Quinn et al. demonstrated that strong activation of innate immune gene expression profiles in the draining lymph nodes (dLNs) correlated with limiting Ad-mediated transgene expression for many rare or non-human Ad types (33). The Ad vectors with the highest levels of transgene Ad expression in dLNs, Ad5, and chimpanzee Ad vector ChAdV-3, also had the most attenuated IFN induction. The latter vectors were both potently immunogenic following immunization in mice. In agreement with this, similar comparative immunogenicity studies in mice have shown that transgene expression levels within muscle and dLNs are lower following immunization with a chimpanzee Ad vector, ChAdOx1, when compared with Ad5, and that this translates to superior immunogenicity observed with Ad5 (123).

To directly address the question of how persistence of Ag contributes to the induction of the robust immunogenicity of Ad vaccines, Finn et al. constructed an Ad vaccine with a doxycycline-regulated transgene expression cassette (121). By switching off transgene expression at early vs. late time-points post-immunization, the authors confirmed the importance of presence of Ag in expanding and maintaining memory T cell responses up to D30, but showed that the maintenance of memory responses at later time-points (D60) is independent of transgene expression.

As discussed above, it is apparent that a combination of multiple parameters influences the extent of transgene expression. These factors include the receptor usage and cellular tropism of each Ad vector, the presence and accessibility of specific cell types at the site of injection, in addition to differences in the induction of early innate immunity by diverse Ad vectors. Collectively, these parameters shape subsequent adaptive immune responses.

#### Route of Administration

The successful transduction of cells at the site of vaccine administration, and subsequent engagement of defined and desirable PRRs which result in robust transgene expression, depend on the cell type and the specific Ad vector being studied. With this in mind, much of the evidence to date has focused on characterizing the induction of innate immune responses *in vitro* in APCs such as DCs or macrophages, which play important roles in initiating anti-viral immune responses. It is considered that inflammation induced at the injection site, can lead to an influx of APCs (monocytes or DCs) which carry Ag to the dLNs (124). Immature DCs residing at the site of vaccination respond to innate immune signals (i.e., stimulation of TLR pathways) by undergoing maturation, upregulating co-stimulatory molecules and migrating to dLNs where they present Ag to naïve T cells (124). However, non-professional APCs expressing MHC I (125, 126), such as tissue-specific epithelial or endothelial cells, could also contribute to the immune sensing of Ad vectors at the site of injection (39). Therefore, it is clear that the route of vaccine administration (45, 127, 128), the abundance and accessibility of cell types at those locations, as well as the surface expression of suitable entry receptors, will profoundly affect the immunological potency and protective efficacy of a chosen Ad vector.

As a result of the long history of experimental use of Ad vectors as oncolytic agents aimed at treating disseminated metastases, a significant amount of information exists in the literature regarding interactions between Ad vectors, immune cells (28, 77, 129), parenchymal cells (18, 63, 113, 130–134), and stromal cells (130) following *i.v*. delivery of Ad vectors (18, 112, 135). However, *i.v*. vaccination would be impractical for widespread population use and so immunization by *i.m*. or *i.n*. administration is preferable, particularly for vaccination against respiratory pathogens. Unfortunately, less is known about the precise interactions which occur at the site of injection or within dLNs *in vivo* following *i.m*. or *i.n*. immunization with Ads in animal models, and these questions are even more difficult to address in humans, without the use of invasive procedures (such as fine needle aspirates of lymph nodes) (136).

##### Intramuscular delivery

The cell types present when vaccine is administered *i.m*. include myocytes, skeletal muscle cells, fibroblasts and endothelial cells, with APCs such as DCs or macrophages representing a minority when compared with the abundance of murine skeletal muscle cells (87). Early studies by Mercier et al. demonstrated that transduction of different cell types can modulate the outcome and phenotype of the humoral immune response following Ad vaccination. The authors transduced DCs (professional APCs), myoblasts (progenitor cells which give rise to muscle cells) and endothelial cells *ex vivo* with Ad5 expressing a model antigen β-galactosidase (β-gal) (87), and vaccinated mice *i.m*. with the Ad-transduced cells. The authors found that all transduced cell types elicited humoral immune responses to the β-gal transgene to a similar extent (albeit with differences in their temporal kinetics), but that the IgG isotype subclass profile differed. Injection of transduced DCs or endothelial cells resulted in production of Ag-specific Abs which were exclusively IgG_2a_, whereas vaccination with Ad-transduced myoblasts elicited a more balanced Ab response with equivalent IgG_1_:IgG_2a_. Interestingly, only mice immunized with Ad-transduced DCs elicited robust CD8^+^ T cell responses, as did vaccination with control virus Ad-β-gal, suggesting that Ad interactions with different cell types *in vivo* could influence divergent arms of the adaptive immune response.

**Figure 4:**
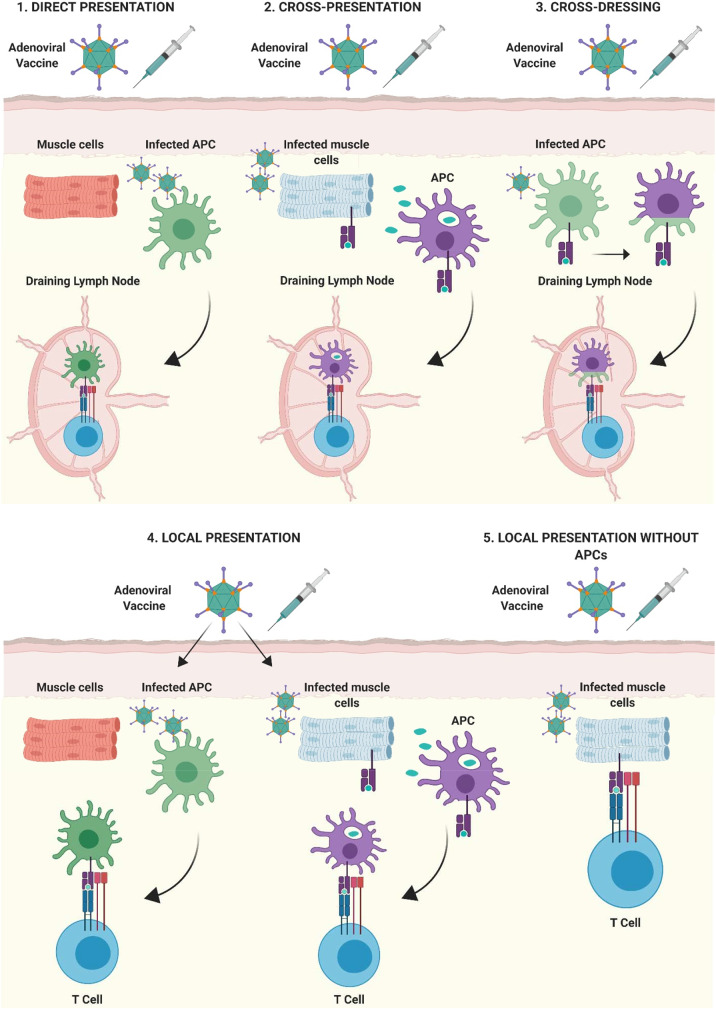
Mechanisms of antigen presentation after intramuscular immunization with adenoviral vectored vaccines. **(1)** *Direct-presentation:* Adenoviral vaccine transduces APCs at the site of injection. APCs migrate to draining lymph nodes (dLNs) where they present processed vaccine antigen to T cells. **(2)** *Cross-presentation:* Vaccine antigen debris from Ad vaccine transduced cells is phagocytosed by professional APCs at the site of injection, transferred to dLNs by APCs and presented to lymphocytes. **(3)** *Cross-dressing:* Peptide:MHC complexes from Ad-transduced APCs may be transferred to naïve APCs by a process of membrane gnawing called trogocytosis. **(4)** APCs present at, or infiltrating into the site of injection, can present antigen directly to T lymphocytes. **(5)** Non-professional APCs such as parenchymal cells at the site of injection (muscle cells shown as an example) can present antigenic peptide on MHC I to infiltrating CD8^+^ T lymphocytes, outside of secondary lymphoid organs. Figure is updated from Coughlan et al. (108) and Holst and Thomsen (138) and was created with ©BioRender - biorender.com.

Bassett et al. also demonstrated that Ag presentation by non-lymphoid cells, in cooperation with hematopoietic APCs, contributes to the kinetics of primary CD8^+^ T cell expansion, the maintenance of memory responses and to the functional phenotype of the cellular immune response following Ad vaccination (39). Through a series of investigations, the authors showed that although dLNs act as the site of immunological priming in response to Ad vaccination, primary expansion of the Ag-specific CD8^+^ T cell response requires a source of sustained Ag expression outside of dLNs (39). They hypothesized that this cell type was of non-hematopoietic origin, due to their prior findings that a radioresistant population of cells was capable of priming CD8^+^ T cell responses in mice leukopenic mice (137). Therefore, it is feasible that several modes of Ag presentation take place following *i.m*. immunization with Ad vectors, all of which contribute to different facets of the ensuing immune response. These interactions are summarized in Figure 4, showing that Ag presentation could take place not only within dLNs, but also at the site of injection, with or without the involvement of professional APCs.

It is important to note that the majority of detailed mechanistic studies into the immunogenicity of Ad vectors have been performed using Ad5-based vaccines and as such, similar information regarding the *in vivo* cellular tropism of rare species or non-human Ad vectors, is much more limited (123). It will therefore be crucial to outline the precise factors which confer a hierarchy of potency between Ad vectors in the future, as many Ad vectors are already under clinical investigation, or are advancing rapidly into clinical trials. This improved knowledge would allow us to engineer optimal platform vectors which combine multiple attributes associated with potent immunogenicity and long-lived protective efficacy.

##### Aerosolized or intranasal delivery

Mucosal *i.n*. or aerosolized (*a.e*.) delivery of Ad vectors is particularly attractive for the development of vaccines against respiratory pathogens (108, 139, 140). Ad vectors are capable of eliciting both humoral and cellular immune responses following *i.n*. (4, 140–142) and *a.e*. (143) vaccination in animal models, both in the bronchoalveolar lavage (BAL) and within the lung interstitium (15, 144). Many wildtype adenoviruses are common respiratory pathogens (i.e., HAdV-C5, HAdV-E4), highlighting their potential suitability for targeting vaccine Ag to mucosal surfaces within the respiratory tract. However, several chimpanzee Ad vectors, which are not associated with respiratory infections, have displayed superior immunogenicity to Ad5 when administered by the *i.n*. route in mice, including a ChAdV-68-based vaccine against pulmonary tuberculosis (TB) (144) and a ChAdV-7 vaccine against *Pseudomonas aeruginosa* (145).

A major limitation in the application of rAd vaccines to mucosal respiratory surfaces may be the rapid scavenging and degradation of Ad vaccine vector particles by tissue resident alveolar macrophages in the lung, as it has previously been estimated that ~70% of Ad vector genomes are lost within the first 24h following lung delivery in mice (intratracheal) (66). Furthermore, alveolar macrophages were found to be the predominant cell type responsible for initial inflammatory cytokine induction (68). However, depending on the balance of innate immune stimulation and the retention of a certain level of transgene expression within the respiratory tract, these interactions could be beneficial for the induction of subsequent adaptive immune responses.

Alveolar macrophages may play a role in trafficking to dLNs to facilitate Ag priming, or the inflammatory cytokines they release could signal the recruitment of lymphoid cells which could further potentiate immune responses to Ad-encoded transgene Ag. The many studies which have confirmed the induction of protective immunity following *i.n*. vaccination with Ad vectors support this possibility, but the precise mechanistic factors which underlie these effects and the specific cell types which contribute to vaccine efficacy are not extensively described. As differences in the phenotype and trafficking potential of CD8^+^ T cells has been observed when comparing *i.m*. vs. *i.n*. vaccination of mice with Ad vectors, it will be important to identify the optimal Ad vaccine platform which elicits the correct correlates of protection for a specific disease target, before deciding on the ideal route of vaccine administration (128).

#### Structural Basis for Immune Activation by Ad Particles

The ability to engage and activate multiple, diverse innate immune signaling pathways simultaneously (excluding Type I IFN) can allow rAd vectors to act as an effective self-adjuvant, relative to other vaccination platforms (109, 110, 146). These attributes suggest that Ad virions themselves possess several PAMPs. In fact, it is considered that the native receptor determinants or entry factors of a particular Ad vector may, at least in part, regulate innate immune activation. In support of this, binding of the fiber knob domain to CAR is sufficient to activate p38MAPK, p44/42MAPK (ERK1/2), and NF-κB pathways (147), resulting in the transcription of pro-inflammatory cytokines both *in vitro* and *in vivo* (148). Similarly, Ad vectors which are capable of using CD46 as a receptor have been shown to display preferential activation of TLR9 to CAR-binding Ad vectors *in vitro* (61). However, CD46 distribution is limited to the expression in the testes in mice, rats and guinea pigs, in contrast to its widespread expression in humans (149). Therefore, this likely has little contribution to the immunological potency of CD46-using Ad vectors in murine models.

The fiber knob domain of Ad5-based vectors has been shown to contribute to the maturation of murine DCs, as recombinant knob protein, full-length fiber protein and penton capsomers (penton plus fiber), but not hexon or penton alone, were capable of exerting this effect (90). In support of this, Teigler et al. previously suggested that fiber-receptor interactions were important for triggering innate immune responses to rare species Ad vectors (26). Furthermore, studies using HAdV-B35 vectors pseudotyped with the fiber knob from Ad5 were shown to be more immunogenic in mice and NHP, suggesting that the fiber knob domain could contribute substantially to immunological potency (150). However, similar studies in which the entire fiber and penton RGD motif from rAd5 were swapped into chimpanzee Ad vector ChAdOx1, failed to increase the immunogenicity of ChAdOx1 relative to Ad5, suggesting that factors beyond fiber/penton capsomer interactions confer immune responses to ChAdOx1 (123).

More recently it has been shown that a member of the vitamin-K dependent protein family, growth arrest specific protein (Gas6), can bind differentially to the fiber of rAd vectors (151), interacting with a putative epitope which is outside of the fiber knob domain, such as the fiber shaft. Interestingly, Gas6 bound efficiently to the fiber of CAR-binding Ad5, without affecting its ability to enter cells, but significantly reduced the induction of type I IFNs, resulting in prolonged transgene expression. Conversely, Gas6 failed to bind to the fibers of non-Ad5 and non-CAR binding viruses, such as a species D Ad vectors HAdV-D28, which has been shown to display reduced immunogenicity in mice when compared with rAd5 (152). In support of this, HAdV-D28 has been shown to elicit robust IFN-α and had increased stimulation of IFN-responsive genes when compared with Ad5 (44). Importantly, immunization of IFNR1^−/−^ mice with HAdV-D28 resulted in robust cellular immune responses, comparable to Ad5 (44). These data support the hypothesis by several other groups that Ad vectors which trigger robust type I IFN responses exhibit reduced transgene expression and impaired vaccine immunogenicity (33, 120). Therefore, one strategy to overcome stimulation of IFN-α could be to identify the precise amino acid binding epitopes for Gas6 and genetically engineer this region into the fiber of non-Gas6 binding Ad vectors in an effort to increase their immunogenicity (151).

In addition to interactions with CAR, interactions between the RGD motif within the penton base and cellular integrins can contribute to the induction of innate immune signaling pathways both *in vitro* (153) and *in vivo* (77). Following *i.v*. administration of Ad5, interactions between the β3 subunit of cellular integrins and the RGD motif on MARCO^+^ marginal zone macrophages in the spleen were required for triggering IL-1α-dependent innate immune signaling in mice (77). However, it is unclear how these interactions affect immune responses following immunization by other routes of administration. Finally, the Ad virion itself and its major capsid protein, the hexon, has been described as a potent adjuvant, capable of activating CD4^+^ and CD8^+^ cellular immune responses to a model immunogen mixture in mice (146). Although the precise mechanism underlying this effect has not been identified, subsequent studies have suggested that the HVR regions, particularly HVR1, are involved in enhancing interactions with scavenging macrophages (76, 78).

#### Novel Approaches to Increasing the Immunological Potency of Ad Vectors

One approach to identifying lead vaccine candidates is to perform head-to-head comparisons of immunogenicity in animal models to identify the most immunogenic vectors, followed by detailed and systematic experimental studies to try to identify the underlying mechanisms which contribute to immunological potency (i.e., transgene expression magnitude and duration, innate immune stimulation). However, in the interim, efforts are ongoing to try to maximize immune recognition of the Ad encoded Ag, in an effort to compensate for the reduced immunogenicity associated with certain Ad vectors which are otherwise an ideal platform (i.e., low seroprevalence, high titer growth, stability). Furthermore, this type of modification could permit dose-sparing, which would have cost saving effects, as well as minimizing any vector-induced reactogenicity, without compromising on immunogenicity. Such approaches include the use of molecular or genetic adjuvants, namely in the form of fusion proteins with the vaccine antigen of interest, which help to potentiate immunogenicity by enhancing Ag presentation or dissemination [reviewed in detail by Neukirch et al. (154)].

One such approach which has previously been described includes the generation of fusion-Ag cassettes which enhance MHC presentation. This can be achieved by fusing vaccine Ag to β-microglobulin for enhanced MHC I presentation (155), or to the invariant chain of MHC II (156–159). These approaches were capable of enhancing antigen-specific CD8^+^ T cells responses in mice following immunization with an Ad vector encoding the Ii or β2-microglobulin fusion antigen. In a separate approach, we recently have shown that encoding vaccine Ag cassettes in which a model antigen, enhanced green fluorescent protein (EGFP), is fused to a protein domain known to tether proteins to the surface of extracellular vesicles (EVs), can dramatically improve the humoral immunogenicity of both Ad5 and chimpanzee Ad vector ChAdOx1 in mice following *i.m*. and *i.n* vaccination. EVs play important roles in regulating immune responses and are conveyors of cellular communication signals (15). As EVs are frequently implicated in host-pathogen interactions and have been shown to transfer antigenic material to APCs (81, 82), we reasoned that directed targeting of a vaccine Ag to their surface could potentiate immune responses. Although cellular immunity was largely unaffected, Ag-specific humoral immune responses were increased up to 400-fold when compared with unmodified EGFP (15). The choice of molecular or genetic adjuvant will depend on the predicted correlates of protection for a specific disease target: in certain cases, a robust humoral immune response will be more important than a cellular immune response. Further to this, some adjuvanting technologies will work for one Ag but not for another, and structural constraints may limit the ability to modify complex, multimeric Ags. This will require the optimization and selection of different components to be combined and assembled into customized Ad vectors which are tailored to specific pathogens.

### Clinical Evaluation of Ad Vaccines for Infectious Disease; Challenges; and Advances

#### Challenges

A number of promising animal studies solidified the appeal of Ad5 as a platform vaccine vector for infectious diseases. In particular, a study by Sullivan et al. demonstrated that a single-shot, low dose (1 × 10^10^ vp) immunization with Ad5 expressing Ebola virus glycoprotein (GP) could provide 100% protection from challenge in NHPs (160). Similar studies using Ad5 based vectors expressing SIV gag demonstrated its superiority to plasmid DNA or modified vaccinia Ankara (MVA) in attenuating viremia following virus challenge (12). On the basis of these promising early results, a multicenter Phase II clinical trial called the Merck STEP study was initiated, in which a vaccine composed of a mixture of Ad5 vectors expressing HIV-1 *gag, pol*, and *nef* genes was administered to participants at high risk for HIV-1 acquisition (161, 162). Phase I safety and immunogenicity studies in healthy, HIV-seronegative adults showed that this vaccine could elicit antigen-specific IFN-γ ELISpot responses in both Ad5 seronegative and seropositive individuals (163). However, the Phase II study was terminated prematurely due to futility and failure to meet pre-defined endpoints: an interim analysis determined that the vaccine would not be efficacious in preventing HIV-1 infection, or in reducing viral-load setpoint in seroconverters, despite eliciting T cell responses in most participants (162, 163). Subsequent to this, a *post-hoc* analysis of the study suggested an association between vaccination with the Ad5 vaccine and increased acquisition of HIV-1 (161). On multivariate analyses, this increase was largely restricted to a defined sub-set of participants: uncircumcised men with high baseline antibody titers against Ad5. Several hypotheses were proposed to explain the increase in HIV-1 acquisition, including the formation of immune complexes (IC) between anti-Ad5 antibodies and DCs, which were shown to enhance HIV-1infection of T cells in DC-T cell co-cultures *in vitro* (164). An alternative hypothesis suggested that Ad5 immunization induced the expansion of Ad-specific memory CD4^+^ T cells which upregulate CCR5 expression and/or homing markers for mucosal sites, thereby increasing the pool of HIV-1 susceptible cells at the site of infection (165). Although the latter hypothesis has been challenged (166), the precise mechanisms underlying the increased acquisition of HIV-1 in the Merck Step trial remain inconclusive (166). However, it is important to note that the effects were shown to wane over time (167). Nonetheless, this outcome led to a dampening in enthusiasm for the broad application of Ad5-based vaccines for major infectious diseases, prompting the investigation of novel rare species human or non-human Ad viruses as alternative vaccine platforms.

#### Advances

Several rare species or non-human Ad vaccines are now leading the way in human clinical trials, namely species C vector HAdV-C6 and species D vector HAdV-26, as well as chimpanzee Ad vectors ChAdV-3, PanAdV-3, ChAdV-63, and ChAdOx1, which cluster phylogenetically with species C or E human Ads (see Table 1). Many of these vector candidates had previously been identified in animal studies as being potently immunogenic, and in some cases were comparable to the potency of Ad5 [(5, 168); Table 2]. In particular, HAdV-C6 and ChAdV-3 appear to possess attributes which make them an attractive platform vector (22%, 12% seroprevalence, respectively) (2, 5), and as a result, have been developed for clinical testing as vaccines against major global pathogens, hepatitis C virus (HCV) (2) and HIV-1 (169). Both HAdV-C6 and ChAdV-3 were shown to be safe and immunogenic in humans when used as a vaccine to elicit immunity against HCV, although HAdV-C6 appeared to be superior in its ability to cellular immune responses with increased magnitude and breadth at lower doses (i.e., 5 × 10^8^ vp) (2). With regard to ChAdV-3, part of its appeal includes its ability to elicit long-lived cellular and humoral immune responses directed toward the encoded vaccine antigen. Immunization of NHPs with ChAdV-3 expressing HIV *gag* resulted in cellular immune responses which persisted for more than 5 years (5). A heterologous boost with PanAdV-3 at week 299 facilitated an expansion of gag-specific IFN-γ secreting T cells, in addition to boosting antibodies to HIV-1 gag. As such, there is broad interest in using these rare vectors in heterologous prime:boost vaccination regimens in an effort to confer long-lived protective immunity against challenging pathogens. In support of this, a Phase I clinical trial to test the HAdV-C6 and ChAdV-3 vectors expressing HCV antigens demonstrated that heterologous boost immunizations resulted in long-lived, polyfunctional effector, and central memory T cell responses which were sustained for up to 1 year in humans (2).

PanAdV-3 expressing Respiratory Syncytial Virus (RSV) fusion (F), nucleocapsid (N), and matrix (M2-1) antigens has also been tested in humans following *i.n*. and *i.m*. administration (37). Neutralizing antibodies to RSV F were increased following *i.m.*, but not *i.n*. prime immunization. Similar trends were observed for antigen-specific T cell responses to the vaccine inserts, although increases were minimal following PanAdV-3 *i.m*. prime only. This vaccine was also evaluated in older adults (60–75 years) who are at increased risk of severe RSV disease, with similar results (170). Immune responses elicited by PanAdV-3 were improved upon boosting with an MVA vector which also encoded the RSV transgene antigen. ChAdV-63, also identified as a clinically viable Ad vector with low seroprevalence which displayed protective efficacy in animal studies (Table 2), has been shown to be safe and immunogenic in children and adults (171–174). When ChAdV-63 has been used in a prime:boost vaccination regimen with MVA expressing malaria antigens, promising efficacy was observed in UK and Kenyan adults (175, 176), but has recently been associated with disappointing efficacy in field trials in young children in Burkina Faso, a highly endemic malaria transmission region (177). ChAdOx-1 is a species E chimpanzee Ad vector developed by the Jenner Institute at University of Oxford which has been tested clinically as a vaccine for influenza virus as a standalone vector or for use in prime:boost with MVA (1, 3), and for several other infectious disease targets such as Chikungunya Fever (NCT03590392), malaria (NCT03203421), and tuberculosis (NCT01829490). Numerous additional trials are currently ongoing or actively recruiting participants, including a recently initiated study to test a novel vaccine for COVID-19 (NCT04324606).

In addition to vectors derived from NHPs, promising advances have been the development of HAdV-D26 vectors. With a number of clinical trials registered on ClinicalTrials.gov, this platform has advanced into clinical studies as a vaccine against Ebola virus (178–180), RSV (181), HIV-1 (6, 182, 183), and has also very recently been announced as a candidate vectored vaccine against COVID-19. The first-in-human testing of HAdV-D26 expressing HIV-1 Env demonstrated that the vaccine was safe and well-tolerated and elicited Env-specific antibodies and antigen-specific ELISpot responses in all participants (182). Although HIV-1 specific neutralizing antibodies were not detected, the study reported multi-functional readouts for the non-neutralizing antibodies elicited, including effector functions such as antibody-dependent cell-mediated phagocytosis (ADCP), antibody-dependent cell-mediated virus inhibition (ADCVI) (183). This vaccine platform was subsequently improved upon by encoding polyvalent “mosaic” HIV-1 antigens, Env, Gag and Pol, representing computationally optimized sequences aimed at maximizing recognition of T cell epitopes (184). Evaluation of the HAdV-D26 platform in various prime:boost regimens is ongoing and preliminary data suggest that it is immunogenic, capable of eliciting Env-specific antibodies which exhibit ADCP and cellular immune responses out to week 50. Importantly, these assays were found to be correlates of protection in a parallel SHIV challenge model in rhesus monkeys (*Macaca mulatta*) (6). The HAdV-D26 mosaic HIV-1 vaccine is currently in Phase IIb efficacy studies in sub-saharan Africa (NCT03060629).

### In Conclusion

Based on the literature, it appears that Ad vectors derived from species C, D, or E are most likely to be immunogenic vectors. Requirements for selecting specific vectors will vary depending on whether the required application is as a stand-alone vaccine or as part of a prime:boost regimen. For standalone vaccination regimens aimed at eliciting a rapid, protective response during an emerging pandemic, the magnitude of response to an identified correlate of protection following a single shot is crucial. Secondary considerations for an evolving pandemic scenario would be rapid immunogenicity at a low dose, and the capacity for lyophilization or stabilization, to facilitate dose-sparing, vaccine cost-effectiveness and pandemic preparedness. In this regard, of the Ad vectors evaluated to date, ChAdV-3 and HAdV-C6 appear promising. For protection against more complex pathogens which require long-lived polyfunctional responses, or sustained humoral immunity with extensive breadth (i.e., universal influenza vaccine, HIV-1 or HCV), heterologous prime:boost vaccination regimens should be evaluated using diverse Ad vectors, or Ads in combination with MVA or protein based immunogens at different intervals. It is difficult to predict which Ad vectors should take precedence as the encoded antigen will need to be tailored to elicit the correct phenotype of immunity against a defined correlate of protection for each specific disease target. However, underpinning the evaluation of all Ad-based vaccines in pre-clinical animal studies, should be the inclusion of species C Ad5 vector controls to represent a benchmark of immunological potency. In addition, Ad vaccine candidates should be compared at several doses to evaluate the maintenance of vaccine potency upon dose de-escalation. The field should also make efforts to improve our understanding of the basic biology of many of these novel Ad vectors, as insights into the receptor usage, interactions of Ad vectors with different cell types following immunization and subsequent stimulation of differential innate signaling pathways will all impact on their downstream immunogenicity and ability to confer protective efficacy. Unfortunately, one major challenge in performing these types of head-to-head comparisons is the lack of widespread availability of many of these rare species or non-human Ad vectors to academic investigators, as many of these are being developed by large pharmaceutical companies.

## Summary

It is clear that a hierarchy exists in the immunological potency observed between rare species human and non-human Ad vectors in various animal species. As outlined above, Ad vector immunogenicity is most likely dependent on a complex combination of factors, rather than any particular factor in isolation. An ideal Ad vaccine platform will combine the following attributes; (i) low seroprevalence in humans, (ii) robust immunogenicity with potential for dose-sparing, and (iii) suitable manufacturing characteristics (i.e., growth to high titers, genome stability). Better understanding of the mechanisms which define effective vaccines, will enable us to design and customize improvements to existing Ad vaccine vectors, enhancing their potential for future clinical use.

## Author Contributions

LC: writing and editing—original and final draft.

## Conflict of Interest

The author declares that the research was conducted in the absence of any commercial or financial relationships that could be construed as a potential conflict of interest.

## References

[1] Antrobus RD Coughlan L Berthoud TK Dicks MD Hill AV Lambe T. Clinical assessment of a novel recombinant simian adenovirus ChAdOx1 as a vectored vaccine expressing conserved Influenza A antigens. Mol Ther. (2014). 22:668–74. 10.1038/mt.2013.284 24374965 PMC3944330

[2] Barnes E Folgori A Capone S Swadling L Aston S Kurioka A. Novel adenovirus-based vaccines induce broad and sustained T cell responses to HCV in man. Sci Transl Med. (2012). 4:115ra111. 10.1126/scitranslmed.3003155 22218690 PMC3627207

[3] Coughlan L Sridhar S Payne R Edmans M Milicic A Venkatraman N. Heterologous two-dose vaccination with simian adenovirus and poxvirus vectors elicits long-lasting cellular immunity to influenza virus A in healthy adults. EBioMedicine. (2018). 29:146–54. 10.1016/j.ebiom.2018.02.011 29519670 PMC5926543

[4] Green CA Scarselli E Voysey M Capone S Vitelli A Nicosia A. Safety and immunogenicity of novel respiratory syncytial virus (RSV) vaccines based on the RSV viral proteins F, N and M2-1 encoded by simian adenovirus (PanAd3-RSV) and MVA (MVA-RSV); protocol for an open-label, dose-escalation, single-centre, phase. 1 clinical trial in healthy adults. BMJ Open. (2015) 5:e008748. 10.1136/bmjopen-2015-008748 26510727 PMC4636663

[5] Colloca S Barnes E Folgori A Ammendola V Capone S Cirillo A. Vaccine vectors derived from a large collection of simian adenoviruses induce potent cellular immunity across multiple species. Sci Transl Med. (2012) 4:115ra112. 10.1126/scitranslmed.3002925 22218691 PMC3627206

[6] Barouch DH Tomaka FL Wegmann F Stieh DJ Alter G Robb ML. Evaluation of a mosaic HIV-1 vaccine in a multicentre, randomised, double-blind, placebo-controlled, phase 1/2a clinical trial (APPROACH) and in rhesus monkeys (NHP 13-19). Lancet. (2018) 392:232–43. 10.1016/S0140-6736(18)31364-3 30047376 PMC6192527

[7] Ewer K Rampling T Venkatraman N Bowyer G Wright D Lambe T. A monovalent chimpanzee adenovirus Ebola vaccine boosted with MVA. New Engl J Med. (2016) 374:1635–46. 10.1056/NEJMoa1411627 25629663 PMC5798586

[8] Quinn KM Da Costa A Yamamoto A Berry D Lindsay RW Darrah PA. Comparative analysis of the magnitude, quality, phenotype, and protective capacity of simian immunodeficiency virus gag-specific CD8+ T cells following human-, simian-, and chimpanzee-derived recombinant adenoviral vector immunization. J Immunol. (2013) 190:2720–35. 10.4049/jimmunol.1202861 23390298 PMC3594325

[9] Casimiro DR Chen L Fu TM Evans RK Caulfield MJ Davies ME. Comparative immunogenicity in rhesus monkeys of DNA plasmid, recombinant vaccinia virus, and replication-defective adenovirus vectors expressing a human immunodeficiency virus type 1 gag gene. J Virol. (2003) 77:6305–13. 10.1128/jvi.77.11.6305-6313.2003 12743287 PMC154996

[10] Tatsis N Ertl HC. Adenoviruses as vaccine vectors. Mol Ther. (2004) 10:616–29. 10.1016/j.ymthe.2004.07.013 15451446 PMC7106330

[11] Geisbert TW Bailey M Hensley L Asiedu C Geisbert J Stanley D. Recombinant adenovirus serotype 26 (Ad26) and Ad35 vaccine vectors bypass immunity to Ad5 and protect nonhuman primates against Ebolavirus challenge. J Virol. (2011) 85:4222–33. 10.1128/JVI.02407-10 21325402 PMC3126236

[12] Shiver JW Fu TM Chen L Casimiro DR Davies ME Evans RK. Replication-incompetent adenoviral vaccine vector elicits effective anti-immunodeficiency-virus immunity. Nature. (2002) 415:331–5. 10.1038/415331a 11797011

[13] Robinson CM Singh G Lee JY Dehghan S Rajaiya J Liu EB. Molecular evolution of human adenoviruses. Sci Rep. (2013) 3:1812. 10.1038/srep01812 23657240 PMC3648800

[14] Singh G Robinson CM Dehghan S Schmidt T Seto D Jones MS. Overreliance on the hexon gene, leading to misclassification of human adenoviruses. J Virol. (2012) 86:4693–5. 10.1128/JVI.06969-11 22301156 PMC3318657

[15] Bliss CM Parsons AJ Nachbagauer R Hamilton JR Cappuccini F Ulaszewska M. Targeting antigen to the surface of EVs improves the *in vivo* immunogenicity of human and non-human adenoviral vaccines in mice. Mol Ther Methods Clin Dev. (2020) 16:108–25. 10.1016/j.omtm.2019.12.003 31934599 PMC6953706

[16] Zhang W Fu J Liu J Wang H Schiwon M Janz S. An engineered virus library as a resource for the spectrum-wide exploration of virus and vector diversity. Cell Rep. (2017) 19:1698–709. 10.1016/j.celrep.2017.05.008 28538186

[17] Russell WC. Adenoviruses: update on structure and function. J Gen Virol. (2009) 90:1–20. 10.1099/vir.0.003087-0 19088268

[18] Coughlan L Alba R Parker AL Bradshaw AC McNeish IA Nicklin SA. Tropism-modification strategies for targeted gene delivery using adenoviral vectors. Viruses. (2010) 2:2290–355. 10.3390/v2102290 21994621 PMC3185574

[19] Zhang W Fu J Ehrhardt A. Novel vector construction based on alternative adenovirus types via homologous recombination. Hum Gene Ther Methods. (2018) 29:124–34. 10.1089/hgtb.2018.044 29756505

[20] Duffy MR Alonso-Padilla J John L Chandra N Khan S Ballmann MZ. Generation and characterization of a novel candidate gene therapy and vaccination vector based on human species D adenovirus type 56. J Gen Virol. (2018) 99:135–47. 10.1099/jgv.0.000978 29154744

[21] Roy S Gao G Lu Y Zhou X Lock M Calcedo R. Characterization of a family of chimpanzee adenoviruses and development of molecular clones for gene transfer vectors. Human Gene Ther. (2004) 15:519–30. 10.1089/10430340460745838 15144581

[22] Kovesdi I Hedley SJ. Adenoviral producer cells. Viruses. (2010) 2:1681–703. 10.3390/v2081681 21994701 PMC3185730

[23] Gurwith M Lock M Taylor EM Ishioka G Alexander J Mayall T. Safety and immunogenicity of an oral, replicating adenovirus serotype 4 vector vaccine for H5N1 influenza: a randomised, double-blind, placebo-controlled, phase 1 study. Lancet Infect Dis. (2013) 13:238–50. 10.1016/S1473-3099(12)70345-6 23369412 PMC3576519

[24] Liebowitz D Gottlieb K Kolhatkar NS Garg SJ Asher JM Nazareno J. Efficacy, immunogenicity, and safety of an oral influenza vaccine: a placebo-controlled and active-controlled phase 2 human challenge study. Lancet Infect Dis. (2020) 20:435–44. 10.1016/S1473-3099(19)30584-5 31978354

[25] Sebastian S Lambe T. Clinical advances in viral-vectored influenza vaccines. Vaccines. (2018) 6:29. 10.3390/vaccines6020029 29794983 PMC6027524

[26] Teigler JE Iampietro MJ Barouch DH. Vaccination with adenovirus serotypes 35, 26, and 48 elicits higher levels of innate cytokine responses than adenovirus serotype 5 in rhesus monkeys. J Virol. (2012) 86:9590–8. 10.1128/JVI.00740-12 22787208 PMC3446581

[27] Dicks MD Spencer AJ Edwards NJ Wadell G Bojang K Gilbert SC. A novel chimpanzee adenovirus vector with low human seroprevalence: improved systems for vector derivation and comparative immunogenicity. PLoS ONE. (2012) 7:e40385. 10.1371/journal.pone.0040385 22808149 PMC3396660

[28] Coughlan L Bradshaw AC Parker AL Robinson H White K Custers J. Ad5:Ad48 hexon hypervariable region substitutions lead to toxicity and increased inflammatory responses following intravenous delivery. Mol Ther. (2012). 20:2268–81. 10.1038/mt.2012.162 22929662 PMC3514487

[29] Abbink P Lemckert AA Ewald BA Lynch DM Denholtz M Smits S. Comparative seroprevalence immunogenicity of six rare serotype recombinant adenovirus vaccine vectors from subgroups B and D. J Virol. (2007) 81:4654–663. 10.1128/JVI.02696-06 17329340 PMC1900173

[30] Mennechet FJD Paris O Ouoba AR Salazar Arenas S Sirima SB Takoudjou Dzomo GR. A review of 65 years of human adenovirus seroprevalence. Expert Rev Vaccines. (2019) 18:597–613. 10.1080/14760584.2019.1588113 31132024

[31] Sayedahmed EE Hassan AO Kumari R Cao W Gangappa S York I. A bovine adenoviral vector-based H5N1 influenza -vaccine provides enhanced immunogenicity and protection at a significantly low dose. Mol Ther Methods Clin Dev. (2018) 10:210–22. 10.1016/j.omtm.2018.07.007 30101154 PMC6082999

[32] Xiang ZQ Yang Y Wilson JM Ertl HC. A replication-defective human adenovirus recombinant serves as a highly efficacious vaccine carrier. Virology. (1996) 219:220–7. 10.1006/viro.1996.0239 8623532

[33] Quinn KM Zak DE Costa A Yamamoto A Kastenmuller K Hill BJ. Antigen expression determines adenoviral vaccine potency independent of IFN and STING signaling. J Clin Invest. (2015) 125:1129–46. 10.1172/JCI78280 25642773 PMC4362254

[34] Yang TC Dayball K Wan YH Bramson J. Detailed analysis of the CD8+ T-cell response following adenovirus vaccination. J Virol. (2003) 77:13407–11. 10.1128/jvi.77.24.13407-13411.2003 14645597 PMC296052

[35] Sullivan NJ Hensley L Asiedu C Geisbert TW Stanley D Johnson J. CD8+ cellular immunity mediates rAd5 vaccine protection against Ebola virus infection of nonhuman primates. Nat Med. (2011) 17:1128–31. 10.1038/nm.2447 21857654

[36] Tatsis N Fitzgerald JC Reyes-Sandoval A Harris-McCoy KC Hensley SE Zhou D. Adenoviral vectors persist *in vivo* and maintain activated CD8+ T cells: implications for their use as vaccines. Blood. (2007) 110:1916–23. 10.1182/blood-2007-02-062117 17510320 PMC1976365

[37] Green CA Scarselli E Sande CJ Thompson AJ de Lara CM Taylor KS. Chimpanzee adenovirus- and MVA-vectored respiratory syncytial virus vaccine is safe and immunogenic in adults. Sci Transl Med. (2015). 7:300ra126. 10.1126/scitranslmed.aac5745 26268313 PMC4669850

[38] Fejer G Freudenberg M Greber UF Gyory I. Adenovirus-triggered innate signalling pathways. Eur J Microbiol Immunol. (2011) 1:279–88. 10.1556/EuJMI.1.2011.4.3 24516734 PMC3918130

[39] Bassett JD Yang TC Bernard D Millar JB Swift SL McGray AJ. CD8+ T-cell expansion and maintenance after recombinant adenovirus immunization rely upon cooperation between hematopoietic and nonhematopoietic antigen-presenting cells. Blood. (2011) 117:1146–55. 10.1182/blood-2010-03-272336 21088134

[40] Jimenez-Chillaron JC Newgard CB Gomez-Foix AM. Increased glucose disposal induced by adenovirus-mediated transfer of glucokinase to skeletal muscle *in vivo*. FASEB J. (1999) 13:2153–60. 10.1096/fasebj.13.15.2153 10593862

[41] Juillard V Villefroy P Godfrin D Pavirani A Venet A Guillet JG. Long-term humoral and cellular immunity induced by a single immunization with replication-defective adenovirus recombinant vector. Eur J Immunol. (1995) 25:3467–73. 10.1002/eji.1830251239 8566039

[42] Tan WG Jin HT West EE Penaloza-MacMaster P Wieland A Zilliox MJ. Comparative analysis of simian immunodeficiency virus gag-specific effector and memory CD8+ T cells induced by different adenovirus vectors. J Virol. (2013) 87:1359–72. 10.1128/JVI.02055-12 23175355 PMC3554140

[43] Penaloza-MacMaster P Provine NM Ra J Borducchi EN McNally A Simmons NL. Alternative serotype adenovirus vaccine vectors elicit memory T cells with enhanced anamnestic capacity compared to Ad5 vectors. J Virol. (2013) 87:1373–84. 10.1128/JVI.02058-12 23152535 PMC3554181

[44] Johnson MJ Petrovas C Yamamoto T Lindsay RW Lore K Gall JG. Type I IFN induced by adenovirus serotypes 28 and 35 has multiple effects on T cell immunogenicity. J Immunol. (2012) 188:6109–18. 10.4049/jimmunol.1103717 22586038 PMC3370104

[45] Holst PJ Orskov C Thomsen AR Christensen JP. Quality of the transgene-specific CD8+ T cell response induced by adenoviral vector immunization is critically influenced by virus dose and route of vaccination. J Immunol. (2010) 184:4431–9. 10.4049/jimmunol.0900537 20212099

[46] Lord R Parsons M Kirby I Beavil A Hunt J Sutton B. Analysis of the interaction between RGD-expressing adenovirus type 5 fiber knob domains and alphavbeta3 integrin reveals distinct binding profiles and intracellular trafficking. J Gen Virol. (2006) 87:2497–2505. 10.1099/vir.0.81620-0 16894187

[47] Wickham TJ Mathias P Cheresh DA Nemerow GR. Integrins alpha v beta 3 and alpha v beta 5 promote adenovirus internalization but not virus attachment. Cell. (1993) 73:309–19. 10.1016/0092-8674(93)90231-e 8477447

[48] Suomalainen M Luisoni S Boucke K Bianchi S Engel DA Greber UF. A direct and versatile assay measuring membrane penetration of adenovirus in single cells. J Virol. (2013) 87:12367–79. 10.1128/JVI.01833-13 24027314 PMC3807902

[49] Burckhardt CJ Suomalainen M Schoenenberger P Boucke K Hemmi S Greber UF. Drifting motions of the adenovirus receptor CAR and immobile integrins initiate virus uncoating and membrane lytic protein exposure. Cell Host Microbe. (2011) 10:105–17. 10.1016/j.chom.2011.07.006 21843868

[50] Greber UF Willetts M Webster P Helenius A. Stepwise dismantling of adenovirus 2 during entry into cells. Cell. (1993) 75:477–86. 10.1016/0092-8674(93)90382-z 8221887

[51] Stewart PL Fuller SD Burnett RM. Difference imaging of adenovirus: bridging the resolution gap between X-ray crystallography and electron microscopy. EMBO J. (1993) 12:2589–99. 8334984 10.1002/j.1460-2075.1993.tb05919.x PMC413505

[52] Wodrich H Henaff D Jammart B Segura-Morales C Seelmeir S Coux O. A capsid-encoded PPxY-motif facilitates adenovirus entry. PLoS Pathog. (2010) 6:e1000808. 10.1371/journal.ppat.1000808 20333243 PMC2841620

[53] Maier O Marvin SA Wodrich H Campbell EM Wiethoff CM. Spatiotemporal dynamics of adenovirus membrane rupture and endosomal escape. J Virol. (2012) 86:10821–828. 10.1128/JVI.01428-12 22855481 PMC3457294

[54] Wiethoff CM Wodrich H Gerace L Nemerow GR. Adenovirus protein VI mediates membrane disruption following capsid disassembly. J Virol. (2005) 79:1992–2000. 10.1128/JVI.79.4.1992-2000.2005 15681401 PMC546575

[55] Gaggar A Shayakhmetov DM Liszewski MK Atkinson JP Lieber A. Localization of regions in CD46 that interact with adenovirus. J Virol. (2005) 79:7503–13. 10.1128/JVI.79.12.7503-7513.2005 15919905 PMC1143628

[56] Sirena D Lilienfeld B Eisenhut M Kalin S Boucke K Beerli RR. The human membrane cofactor CD46 is a receptor for species B adenovirus serotype 3. J Virol. (2004) 78:4454–62. 10.1128/jvi.78.9.4454-4462.2004 15078926 PMC387694

[57] Gaggar A Shayakhmetov DM Lieber A. CD46 is a cellular receptor for group B adenoviruses. Nat Med. (2003) 9:1408–12. 10.1038/nm952 14566335

[58] Wang H Li ZY Liu Y Persson J Beyer I Moller T. Desmoglein 2 is a receptor for adenovirus serotypes 3, 7, 11 and 14. Nat Med. (2011) 17:96–104. 10.1038/nm.2270 21151137 PMC3074512

[59] Baker AT Mundy RM Davies JA Rizkallah PJ Parker AL. Human adenovirus type 26 uses sialic acid-bearing glycans as a primary cell entry receptor. Sci Adv. (2019) 5:eaax3567. 10.1126/sciadv.aax3567 31517055 PMC6726447

[60] Shayakhmetov DM Li ZY Ternovoi V Gaggar A Gharwan H Lieber A. The interaction between the fiber knob domain and the cellular attachment receptor determines the intracellular trafficking route of adenoviruses. J Virol. (2003) 77:3712–23. 10.1128/jvi.77.6.3712-3723.2003 12610146 PMC149506

[61] Iacobelli-Martinez M Nemerow GR. Preferential activation of Toll-like receptor nine by CD46-utilizing adenoviruses. J Virol. (2007) 81:1305–12. 10.1128/JVI.01926-06 17108047 PMC1797540

[62] Maler MD Nielsen PJ Stichling N Cohen I Ruzsics Z Wood C. Key role of the scavenger receptor MARCO in mediating adenovirus infection and subsequent innate responses of macrophages. mBio. (2017) 8:e00670-17. 10.1128/mBio.00670-17 28765216 PMC5539421

[63] Shayakhmetov DM Li ZY Ni S Lieber A. Analysis of adenovirus sequestration in the liver, transduction of hepatic cells, and innate toxicity after injection of fiber-modified vectors. J Virol. (2004) 78:5368–81. 10.1128/jvi.78.10.5368-5381.2004 15113916 PMC400378

[64] Tao N Gao GP Parr M Johnston J Baradet T Wilson JM. Sequestration of adenoviral vector by Kupffer cells leads to a nonlinear dose response of transduction in liver. Mol Ther. (2001) 3:28–35. 10.1006/mthe.2000.0227 11162308

[65] Carey B Staudt MK Bonaminio D van der Loo JC Trapnell BC. PU.1 redirects adenovirus to lysosomes in alveolar macrophages, uncoupling internalization from infection. J Immunol. (2007) 178:2440–7. 10.4049/jimmunol.178.4.2440 17277151

[66] Worgall S Leopold PL Wolff G Ferris B Van Roijen N Crystal RG. Role of alveolar macrophages in rapid elimination of adenovirus vectors administered to the epithelial surface of the respiratory tract. Human Gene Ther. (1997) 8:1675–84. 10.1089/hum.1997.8.14-1675 9322870

[67] Stichling N Suomalainen M Flatt JW Schmid M Pacesa M Hemmi S. Lung macrophage scavenger receptor SR-A6 (MARCO) is an adenovirus type-specific virus entry receptor. PLoS pathog. (2018) 14:e1006914. 10.1371/journal.ppat.1006914 29522575 PMC5862501

[68] Zsengeller Z Otake K Hossain SA Berclaz PY Trapnell BC. Internalization of adenovirus by alveolar macrophages initiates early proinflammatory signaling during acute respiratory tract infection. J Virol. (2000) 74:9655–9667. 10.1128/jvi.74.20.9655-9667.2000 11000238 PMC112398

[69] Manickan E Smith JS Tian J Eggerman TL Lozier JN Muller J. Rapid Kupffer cell death after intravenous injection of adenovirus vectors. Mol Ther. (2006) 13:108–17. 10.1016/j.ymthe.2005.08.007 16198149

[70] Schiedner G Bloch W Hertel S Johnston M Molojavyi A Dries V. A hemodynamic response to intravenous adenovirus vector particles is caused by systemic kupffer cell-mediated activation of endothelial cells. Human Gene Ther. (2003) 14:1631–41. 10.1089/104303403322542275 14633405

[71] Xu Z Tian J Smith JS Byrnes AP. Clearance of adenovirus by Kupffer cells is mediated by scavenger receptors, natural antibodies, and complement. J Virol. (2008) 82:11705–13. 10.1128/JVI.01320-08 18815305 PMC2583672

[72] Cotter MJ Zaiss AK Muruve DA. Neutrophils interact with adenovirus vectors via Fc receptors and complement receptor 1. J Virol. (2005) 79:14622–31. 10.1128/JVI.79.23.14622-14631.2005 16282462 PMC1287577

[73] Zaiss AK Vilaysane A Cotter MJ Clark SA Meijndert HC Colarusso P. Antiviral antibodies target adenovirus to phagolysosomes and amplify the innate immune response. J Immunol. (2009) 182:7058–68. 10.4049/jimmunol.0804269 19454703

[74] Haisma HJ Boesjes M Beerens AM van der Strate BW Curiel DT Pluddemann A. Scavenger receptor A: a new route for adenovirus 5. Mol Pharm. (2009) 6:366–74. 10.1021/mp8000974 19227971

[75] Canton J Neculai D Grinstein S. Scavenger receptors in homeostasis and immunity. Nat Rev. (2013) 13:621–34. 10.1038/nri3515 23928573

[76] Khare R Reddy VS Nemerow GR Barry MA. Identification of adenovirus serotype 5 hexon regions that interact with scavenger receptors. J Virol. (2012) 86:2293–301. 10.1128/JVI.05760-11 22156515 PMC3302413

[77] Di Paolo NC Miao EA Iwakura Y Murali-Krishna K Aderem A Flavell RA. Virus binding to a plasma membrane receptor triggers interleukin-1 alpha-mediated proinflammatory macrophage response *in vivo*. Immunity. (2009) 31:110–21. 10.1016/j.immuni.2009.04.015 19576795 PMC2759279

[78] Alemany R Suzuki K Curiel DT. Blood clearance rates of adenovirus type 5 in mice. J Gen Virol. (2000) 81:2605–9. 10.1099/0022-1317-81-11-2605 11038370

[79] Furumoto K Nagayama S Ogawara K Takakura Y Hashida M Higaki K. Hepatic uptake of negatively charged particles in rats: possible involvement of serum proteins in recognition by scavenger receptor. J Control Rel. (2004) 97:133–41. 10.1016/j.jconrel.2004.03.004 15147811

[80] Dolan BP Gibbs KD Jr. Ostrand-Rosenberg S. Dendritic cells cross-dressed with peptide MHC class I complexes prime CD8+ T cells. J Immunol. (2006) 177:6018–24. 10.4049/jimmunol.177.9.6018 17056526

[81] Thery C Duban L Segura E Veron P Lantz O Amigorena S. Indirect activation of naive CD4+ T cells by dendritic cell-derived exosomes. Nat Immunol. (2002). 3:1156–62. 10.1038/ni854 12426563

[82] Thery C Ostrowski M Segura E. Membrane vesicles as conveyors of immune responses. Nat Rev. (2009) 9:581–93. 10.1038/nri2567 19498381

[83] Denzer K van Eijk M Kleijmeer MJ Jakobson E de Groot C Geuze HJ. Follicular dendritic cells carry MHC class II-expressing microvesicles at their surface. J Immunol. (2000) 165:1259–65. 10.4049/jimmunol.165.3.1259 10903724

[84] Segura E Guerin C Hogg N Amigorena S Thery C. CD8+ dendritic cells use LFA-1 to capture MHC-peptide complexes from exosomes *in vivo*. J Immunol. (2007) 179:1489–96. 10.4049/jimmunol.179.3.1489 17641014

[85] Yewdell JW Dolan BP. Immunology: cross-dressers turn on T cells. Nature. (2011) 471:581–2. 10.1038/471581a 21455165 PMC3400133

[86] Lindsay RW Darrah PA Quinn KM Wille-Reece U Mattei LM Iwasaki A. CD8+ T cell responses following replication-defective adenovirus serotype 5 immunization are dependent on CD11c+ dendritic cells but show redundancy in their requirement of TLR and nucleotide-binding oligomerization domain-like receptor signaling. J Immunol. (2010) 185:1513–21. 10.4049/jimmunol.1000338 20610651

[87] Mercier S Gahery-Segard H Monteil M Lengagne R Guillet JG Eloit M. Distinct roles of adenovirus vector-transduced dendritic cells, myoblasts, and endothelial cells in mediating an immune response against a transgene product. J Virol. (2002) 76:2899–911. 10.1128/jvi.76.6.2899-2911.2002 11861857 PMC136003

[88] Korst RJ Mahtabifard A Yamada R Crystal RG. Effect of adenovirus gene transfer vectors on the immunologic functions of mouse dendritic cells. Mol Ther. (2002) 5:307–15. 10.1006/mthe.2002.0538 11863421

[89] Morelli AE Larregina AT Ganster RW Zahorchak AF Plowey JM Takayama T. Recombinant adenovirus induces maturation of dendritic cells via an NF-kappaB-dependent pathway. J Virol. (2000) 74:9617–28. 10.1128/jvi.74.20.9617-9628.2000 11000234 PMC112394

[90] Molinier-Frenkel V Prevost-Blondel A Hong SS Lengagne R Boudaly S Magnusson MK. The maturation of murine dendritic cells induced by human adenovirus is mediated by the fiber knob domain. J Biol Chem. (2003) 278:37175–82. 10.1074/jbc.M303496200 12855705

[91] Bonifaz L Bonnyay D Mahnke K Rivera M Nussenzweig MC Steinman RM. Efficient targeting of protein antigen to the dendritic cell receptor DEC-205 in the steady state leads to antigen presentation on major histocompatibility complex class I products and peripheral CD8+ T cell tolerance. J Exp Med. (2002) 196:1627–38. 10.1084/jem.20021598 12486105 PMC2196060

[92] Johnson TS Mahnke K Storn V Schonfeld K Ring S Nettelbeck DM. Inhibition of melanoma growth by targeting of antigen to dendritic cells via an anti-DEC-205 single-chain fragment variable molecule. Clin Cancer Res. (2008) 14:8169–77. 10.1158/1078-0432.CCR-08-1474 19088032

[93] Pereboev AV Nagle JM Shakhmatov MA Triozzi PL Matthews QL Kawakami Y. Enhanced gene transfer to mouse dendritic cells using adenoviral vectors coated with a novel adapter molecule. Mol Ther. (2004) 9:712–20. 10.1016/j.ymthe.2004.02.006 15120332

[94] Korokhov N de Gruijl TD Aldrich WA Triozzi PL Banerjee PT Gillies SD. High efficiency transduction of dendritic cells by adenoviral vectors targeted to DC-SIGN. Cancer Biol Ther. (2005) 4:289–94. 10.4161/cbt.4.3.1499 15753654

[95] Adams WC Bond E Havenga MJ Holterman L Goudsmit J Karlsson Hedestam GB. Adenovirus serotype 5 infects human dendritic cells via a coxsackievirus-adenovirus receptor-independent receptor pathway mediated by lactoferrin DC-SIGN. J Gen Virol. (2009) 90:1600–10. 10.1099/vir.0.008342-0 19282435 PMC7346604

[96] Lore K Adams WC Havenga MJ Precopio ML Holterman L Goudsmit J. Myeloid and plasmacytoid dendritic cells are susceptible to recombinant adenovirus vectors and stimulate polyfunctional memory T cell responses. J Immunol. (2007) 179:1721–9. 10.4049/jimmunol.179.3.1721 17641038 PMC2365753

[97] Tatsis N Blejer A Lasaro MO Hensley SE Cun A Tesema L. A CD46-binding chimpanzee adenovirus vector as a vaccine carrier. Mol Ther. (2007) 15:608–17. 10.1038/sj.mt.6300078 17228314

[98] Short JJ Pereboev AV Kawakami Y Vasu C Holterman MJ Curiel DT. Adenovirus serotype 3 utilizes CD80 (B7.1) and CD86 (B7.2) as cellular attachment receptors. Virology. (2004) 322:349–59. 10.1016/j.virol.2004.02.016 15110532

[99] Chondronasiou D Eisden T Stam AGM Matthews QL Icyuz M Hooijberg E. Improved induction of anti-melanoma t cells by adenovirus-5/3 fiber modification to target human DCs. Vaccines. (2018) 6:42. 10.3390/vaccines6030042 30022005 PMC6161112

[100] Wilkinson-Ryan I Kim J Kim S Ak F Dodson L Colonna M. Incorporation of porcine adenovirus 4 fiber protein enhances infectivity of adenovirus vector on dendritic cells: implications for immune-mediated cancer therapy. PLoS ONE. (2015) 10:e0125851. 10.1371/journal.pone.0125851 25933160 PMC4416912

[101] Hsu C Boysen M Gritton LD Frosst PD Nemerow GR Von Seggern DJ. *In vitro* dendritic cell infection by pseudotyped adenoviral vectors does not correlate with their *in vivo* immunogenicity. Virology. (2005) 332:1–7. 10.1016/j.virol.2004.11.014 15661134

[102] Vitelli A Folgori A Scarselli E Colloca S Capone S Nicosia A. Chimpanzee adenoviral vectors as vaccines - challenges to move the technology into the fast lane. Expert Rev Vaccines. (2017) 16:1241–52. 10.1080/14760584.2017.1394842 29047309

[103] Vemula SV Mittal SK. Production of adenovirus vectors and their use as a delivery system for influenza vaccines. Expert Opin Biol Ther. (2010). 10:1469–1487. 10.1517/14712598.2010.519332 20822477 PMC2951029

[104] Capelle MAH Babich L van Deventer-Troost JPE Salerno D Krijgsman K Dirmeier U. Stability and suitability for storage and distribution of Ad26.ZEBOV/MVA-BN(R)-Filo heterologous prime-boost Ebola vaccine. Eur J Pharm Biopharm. (2018) 129:215–21. 10.1016/j.ejpb.2018.06.001 29870747

[105] Afkhami S LeClair DA Haddadi S Lai R Toniolo SP Ertl HC. Spray dried human and chimpanzee adenoviral-vectored vaccines are thermally stable and immunogenic *in vivo*. Vaccine. (2017) 35:2916–24. 10.1016/j.vaccine.2017.04.026 28438408

[106] Alcock R Cottingham MG Rollier CS Furze J De Costa SD Hanlon M. Long-term thermostabilization of live poxviral and adenoviral vaccine vectors at supraphysiological temperatures in carbohydrate glass. Sci Transl Med. (2010) 2:19ra12. 10.1126/scitranslmed.3000490 20371486

[107] Wang C Dulal P Zhou X Xiang Z Goharriz H Banyard A. A simian-adenovirus-vectored rabies vaccine suitable for thermostabilisation and clinical development for low-cost single-dose pre-exposure prophylaxis. PLoS Negl Trop Dis. (2018) 12:e0006870. 10.1371/journal.pntd.0006870 30372438 PMC6224154

[108] Coughlan L Mullarkey C Gilbert S. Adenoviral vectors as novel vaccines for influenza. J Pharm Pharmacol. (2015) 67:382–399. 10.1111/jphp.12350 25560474

[109] Rhee EG Blattman JN Kasturi SP Kelley RP Kaufman DR Lynch DM. Multiple innate immune pathways contribute to the immunogenicity of recombinant adenovirus vaccine vectors. J Virol. (2011) 85:315–23. 10.1128/JVI.01597-10 20962088 PMC3014160

[110] Zhu J Huang X Yang Y. Innate immune response to adenoviral vectors is mediated by both Toll-like receptor-dependent and -independent pathways. J Virol. (2007) 81:3170–80. 10.1128/JVI.02192-06 17229689 PMC1866082

[111] Muruve DA Petrilli V Zaiss AK White LR Clark SA Ross PJ. The inflammasome recognizes cytosolic microbial and host DNA and triggers an innate immune response. Nature. (2008) 452:103–7. 10.1038/nature06664 18288107

[112] Atasheva S Shayakhmetov DM. Adenovirus sensing by the immune system. Curr Opin Virol. (2016) 21:109–13. 10.1016/j.coviro.2016.08.017 27639089 PMC5138075

[113] Appledorn DM Patial S McBride A Godbehere S Van Rooijen N Parameswaran N. Adenovirus vector-induced innate inflammatory mediators, MAPK signaling, as well as adaptive immune responses are dependent upon both TLR2 and TLR9 *in vivo*. J Immunol. (2008) 181:2134–44. 10.4049/jimmunol.181.3.2134 18641352

[114] Appledorn DM Patial S Godbehere S Parameswaran N Amalfitano A. TRIF, and TRIF-interacting TLRs differentially modulate several adenovirus vector-induced immune responses. J Innate Immun. (2009) 1:376–88. 10.1159/000207194 20375595 PMC2853581

[115] Lam E Stein S Falck-Pedersen E. Adenovirus detection by the cGAS/STING/TBK1 DNA sensing cascade. J Virol. (2014) 88:974–81. 10.1128/JVI.02702-13 24198409 PMC3911663

[116] Anghelina D Lam E Falck-Pedersen E. Diminished innate antiviral response to adenovirus vectors in cGAS/STING-deficient mice minimally impacts adaptive immunity. J Virol. (2016) 90:5915–27. 10.1128/JVI.00500-16 27076643 PMC4907218

[117] Burdette DL Monroe KM Sotelo-Troha K Iwig JS Eckert B Hyodo M. STING is a direct innate immune sensor of cyclic di-GMP. Nature. (2011) 478:515–8. 10.1038/nature10429 21947006 PMC3203314

[118] Hartman ZC Appledorn DM Amalfitano A. Adenovirus vector induced innate immune responses: impact upon efficacy and toxicity in gene therapy and vaccine applications. Virus Res. (2008) 132:1–4. 10.1016/j.virusres.2007.10.005 18036698 PMC4039020

[119] Hendrickx R Stichling N Koelen J Kuryk L Lipiec A Greber UF. Innate immunity to adenovirus. Human Gene Ther. (2014) 25:265–84. 10.1089/hum.2014.001 24512150 PMC3996939

[120] Hensley SE Cun AS Giles-Davis W Li Y Xiang Z Lasaro MO. Type I interferon inhibits antibody responses induced by a chimpanzee adenovirus vector. Mol Ther. (2007) 15:393–403. 10.1038/sj.mt.6300024 17235319

[121] Finn JD Bassett J Millar JB Grinshtein N Yang TC Parsons R. Persistence of transgene expression influences CD8+ T-cell expansion and maintenance following immunization with recombinant adenovirus. J Virol. (2009) 83:12027–36. 10.1128/JVI.00593-09 19759135 PMC2786755

[122] Yang TC Millar J Groves T Grinshtein N Parsons R Takenaka S. The CD8+ T cell population elicited by recombinant adenovirus displays a novel partially exhausted phenotype associated with prolonged antigen presentation that nonetheless provides long-term immunity. J Immunol. (2006) 176:200–10. 10.4049/jimmunol.176.1.200 16365411

[123] Dicks MD Spencer AJ Coughlan L Bauza K Gilbert SC Hill AV. Differential immunogenicity between HAdV-5 and chimpanzee adenovirus vector ChAdOx1 is independent of fiber and penton RGD loop sequences in mice. Sci Rep. (2015) 5:16756. 10.1038/srep16756 26576856 PMC4649739

[124] Pulendran B Ahmed R. Translating innate immunity into immunological memory: implications for vaccine development. Cell. (2006) 124:849–63. 10.1016/j.cell.2006.02.019 16497593

[125] Thomas S Kolumam GA Murali-Krishna K. Antigen presentation by nonhemopoietic cells amplifies clonal expansion of effector CD8 T cells in a pathogen-specific manner. J Immunol. (2007) 178:5802–11. 10.4049/jimmunol.178.9.5802 17442964

[126] Dzierszinski F Pepper M Stumhofer JS LaRosa DF Wilson EH Turka LA. Presentation of *Toxoplasma gondii* antigens via the endogenous major histocompatibility complex class I pathway in nonprofessional and professional antigen-presenting cells. Infect Immun. (2007) 75:5200–9. 10.1128/IAI.00954-07 17846116 PMC2168266

[127] Lambe T Carey JB Li Y Spencer AJ van Laarhoven A Mullarkey CE. Immunity against heterosubtypic influenza virus induced by adenovirus and MVA expressing nucleoprotein and matrix protein-1. Sci Rep. (2013) 3:1443. 10.1038/srep01443 23485942 PMC3595699

[128] Kaufman DR Bivas-Benita M Simmons NL Miller D Barouch DH. Route of adenovirus-based HIV-1 vaccine delivery impacts the phenotype and trafficking of vaccine-elicited CD8+ T lymphocytes. J Virol. (2010) 84:5986–5996. 10.1128/JVI.02563-09 20357087 PMC2876628

[129] Coughlan L Vallath S Gros A Gimenez-Alejandre M Van Rooijen N Thomas GJ. Combined fiber modifications both to target alpha(v)beta(6) and detarget the coxsackievirus-adenovirus receptor improve virus toxicity profiles *in vivo* but fail to improve antitumoral efficacy relative to adenovirus serotype 5. Human Gene Ther. (2012) 23:960–79. 10.1089/hum.2011.218 22708837

[130] Alba R Bradshaw AC Coughlan L Denby L McDonald RA Waddington SN. Biodistribution and retargeting of FX-binding ablated adenovirus serotype 5. Vectors Blood. (2010) 116:2656–64. 10.1182/blood-2009-12-260026 20610817 PMC2974579

[131] Shayakhmetov DM Gaggar A Ni S Li ZY Lieber A. Adenovirus binding to blood factors results in liver cell infection and hepatotoxicity. J Virol. (2005) 79:7478–91. 10.1128/JVI.79.12.7478-7491.2005 15919903 PMC1143681

[132] Waddington SN McVey JH Bhella D Parker AL Barker K Atoda H. Adenovirus serotype 5 hexon mediates liver gene transfer. Cell. (2008) 132:397–409. 10.1016/j.cell.2008.01.016 18267072

[133] Bradshaw AC Coughlan L Miller AM Alba R van Rooijen N Nicklin SA. Biodistribution and inflammatory profiles of novel penton and hexon double-mutant serotype 5 adenoviruses. J Control Rel. (2012) 164:394–402. 10.1016/j.jconrel.2012.05.025 22626939 PMC3520007

[134] Bradshaw AC Parker AL Duffy MR Coughlan L van Rooijen N Kahari VM. Requirements for receptor engagement during infection by adenovirus complexed with blood coagulation factor X. PLoS Pathog. (2010) 6:e1001142. 10.1371/journal.ppat.1001142 20949078 PMC2951380

[135] Atasheva S Yao J Shayakhmetov DM. Innate immunity to adenovirus: lessons from mice. FEBS Lett. (2019) 593:3461–83. 10.1002/1873-3468.13696 31769012 PMC6928416

[136] Havenar-Daughton C Newton IG Zare SY Reiss SM Schwan B Suh MJ. Normal human lymph node T follicular helper cells and germinal center B cells accessed via fine needle aspirations. J Immunol Methods. (2020) 479:112746. 10.1016/j.jim.2020.112746 31958451 PMC7200018

[137] Grinshtein N Yang TC Parsons R Millar J Denisova G Dissanayake D. Recombinant adenovirus vaccines can successfully elicit CD8+ T cell immunity under conditions of extreme leukopenia. Mol Ther. (2006) 13:270–9. 10.1016/j.ymthe.2005.09.018 16297666

[138] Holst P Thomsen A Harnessing the potential of adenovirus vectored vaccines. In: Viral Gene Therapy. (2011). Available online at: https://www.intechopen.com/books/viral-gene-therapy/harnessing-the-potential-of-adenovirus-vectored-vaccines/

[139] Coughlan L Palese P Overcoming barriers in the path to a universal influenza virus vaccine. Cell Host Microbe. (2018). 24:18–24. 10.1016/j.chom.2018.06.016 30001520

[140] Kim MH Kim HJ Chang J. Superior immune responses induced by intranasal immunization with recombinant adenovirus-based vaccine expressing full-length Spike Protein of Middle East respiratory syndrome coronavirus. PLoS ONE. (2019) 14:e0220196. 10.1371/journal.pone.0220196 31329652 PMC6645677

[141] Jia W Channappanavar R Zhang C Li M Zhou H Zhang S. Single intranasal immunization with chimpanzee adenovirus-based vaccine induces sustained and protective immunity against MERS-CoV infection. Emerg. Microbes Infect. (2019) 8:760–72. 10.1080/22221751.2019.1620083 31130102 PMC6542157

[142] Matchett WE Anguiano-Zarate SS Barry MA. Comparison of systemic and mucosal immunization with replicating Single cycle Adenoviruses. Glob Vaccines Immunol. (2018) 3:128. 10.15761/GVI.1000128 30740532 PMC6368267

[143] Bolton DL Song K Tomaras GD Rao S Roederer M. Unique cellular and humoral immunogenicity profiles generated by aerosol, intranasal, or parenteral vaccination in rhesus macaques. Vaccine. (2017) 35:639–46. 10.1016/j.vaccine.2016.12.008 28041780 PMC5241230

[144] Jeyanathan M Thanthrige-Don N Afkhami S Lai R Damjanovic D Zganiacz A. Novel chimpanzee adenovirus-vectored respiratory mucosal tuberculosis vaccine: overcoming local anti-human adenovirus immunity for potent TB protection. Mucosal Immunol. (2015) 8:1373–87. 10.1038/mi.2015.29 25872483

[145] Krause A Whu WZ Xu Y Joh J Crystal RG Worgall S. Protective anti-Pseudomonas aeruginosa humoral and cellular mucosal immunity by AdC7-mediated expression of the *P. aeruginosa* protein OprF. Vaccine. (2011) 29:2131–9. 10.1016/j.vaccine.2010.12.087 21215829 PMC3061442

[146] Molinier-Frenkel V Lengagne R Gaden F Hong SS Choppin J Gahery-Segard H. Adenovirus hexon protein is a potent adjuvant for activation of a cellular immune response. J Virol. (2002) 76:127–35. 10.1128/jvi.76.1.127-135.2002 11739678 PMC135719

[147] Tamanini A Nicolis E Bonizzato A Bezzerri V Melotti P Assael BM. Interaction of adenovirus type 5 fiber with the coxsackievirus and adenovirus receptor activates inflammatory response in human respiratory cells. J Virol. (2006) 80:11241–54. 10.1128/JVI.00721-06 16956941 PMC1642173

[148] Schoggins JW Nociari M Philpott N Falck-Pedersen E. Influence of fiber detargeting on adenovirus-mediated innate and adaptive immune activation. J Virol. (2005) 79:11627–37. 10.1128/JVI.79.18.11627-11637.2005 16140740 PMC1212603

[149] Miwa T Nonaka M Okada N Wakana S Shiroishi T Okada H. Molecular cloning of rat and mouse membrane cofactor protein (MCP, CD46): preferential expression in testis and close linkage between the mouse Mcp and Cr2 genes on distal chromosome 1. Immunogenetics. (1998) 48:363–71. 10.1007/s002510050447 9799332

[150] Nanda A Lynch DM Goudsmit J Lemckert AA Ewald BA Sumida SM. Immunogenicity of recombinant fiber-chimeric adenovirus serotype 35 vector-based vaccines in mice and rhesus monkeys. J Virol. (2005) 79:14161–8. 10.1128/JVI.79.22.14161-14168.2005 16254351 PMC1280229

[151] Nidetz NF Gallagher TM Wiethoff CM. Inhibition of type I interferon responses by adenovirus serotype-dependent Gas6 binding. Virology. (2018) 515:150–7. 10.1016/j.virol.2017.12.016 29288958 PMC6110086

[152] Kahl CA Bonnell J Hiriyanna S Fultz M Nyberg-Hoffman C Chen P. Potent immune responses and *in vitro* pro-inflammatory cytokine suppression by a novel adenovirus vaccine vector based on rare human serotype 28. Vaccine. (2010) 28:5691–702. 10.1016/j.vaccine.2010.06.050 20600496 PMC2927224

[153] Tibbles LA Spurrell JC Bowen GP Liu Q Lam M Zaiss AK. Activation of p38 and ERK signaling during adenovirus vector cell entry lead to expression of the C-X-C chemokine IP-10. J Virol. (2002) 76:1559–68. 10.1128/jvi.76.4.1559-1568.2002 11799150 PMC135878

[154] Neukirch L Fougeroux C Andersson AC Holst PJ. The potential of adenoviral vaccine vectors with altered antigen presentation capabilities. Expert Rev Vaccines. (2020) 19:25–41. 10.1080/14760584.2020.1711054 31889453

[155] Holst PJ Bartholdy C Stryhn A Thomsen AR Christensen JP. Rapid and sustained CD4(+) T-cell-independent immunity from adenovirus-encoded vaccine antigens. J Gen Virol. (2007) 88:1708–16. 10.1099/vir.0.82727-0 17485530

[156] Mikkelsen M Holst PJ Bukh J Thomsen AR Christensen JP. Enhanced and sustained CD8+ T cell responses with an adenoviral vector-based hepatitis C virus vaccine encoding NS3 linked to the MHC class II chaperone protein invariant chain. J Immunol. (2011) 186:2355–64. 10.4049/jimmunol.1001877 21257961

[157] Spencer AJ Cottingham MG Jenks JA Longley RJ Capone S Colloca S. Enhanced vaccine-induced CD8+ T cell responses to malaria antigen ME-TRAP by fusion to MHC class ii invariant chain. PLoS ONE. (2014) 9:e100538. 10.1371/journal.pone.0100538 24945248 PMC4063960

[158] Jensen S Steffensen MA Jensen BA Schluter D Christensen JP Thomsen AR. Adenovirus-based vaccine against listeria monocytogenes: extending the concept of invariant chain linkage. J Immunol. (2013) 191:4152–64. 10.4049/jimmunol.1301290 24043891

[159] Halbroth BR Sebastian S Poyntz HC Bregu M Cottingham MG Hill AVS. Development of a molecular adjuvant to enhance antigen-specific CD8(+) T cell responses. Sci Rep. (2018) 8:15020. 10.1038/s41598-018-33375-1 30301933 PMC6177389

[160] Sullivan NJ Geisbert TW Geisbert JB Shedlock DJ Xu L Lamoreaux L. Immune protection of nonhuman primates against Ebola virus with single low-dose adenovirus vectors encoding modified GPs. PLoS Med. (2006) 3:e177. 10.1371/journal.pmed.0030177 16683867 PMC1459482

[161] Buchbinder SP Mehrotra DV Duerr A Fitzgerald DW Mogg R Li D. Efficacy assessment of a cell-mediated immunity HIV-1 vaccine (the Step Study): a double-blind, randomised, placebo-controlled, test-of-concept trial. Lancet. (2008) 372:1881–93. 10.1016/S0140-6736(08)61591-3 19012954 PMC2721012

[162] McElrath MJ De Rosa SC Moodie Z Dubey S Kierstead L Janes H. HIV-1 vaccine-induced immunity in the test-of-concept Step Study: a case-cohort analysis. Lancet. (2008) 372:1894–905. 10.1016/S0140-6736(08)61592-5 19012957 PMC2774110

[163] Priddy FH Brown D Kublin J Monahan K Wright DP Lalezari J. Safety and immunogenicity of a replication-incompetent adenovirus type 5 HIV-1 clade B gag/pol/nef vaccine in healthy adults. Clin Infect Dis. (2008) 46:1769–81. 10.1086/587993 18433307

[164] Perreau M Pantaleo G Kremer EJ. Activation of a dendritic cell-T cell axis by Ad5 immune complexes creates an improved environment for replication of HIV in T cells. J Exp Med. (2008) 205:2717–25. 10.1084/jem.20081786 18981239 PMC2585831

[165] Benlahrech A Harris J Meiser A Papagatsias T Hornig J Hayes P. Adenovirus vector vaccination induces expansion of memory CD4 T cells with a mucosal homing phenotype that are readily susceptible to HIV-1. Proc Natl Acad Sci USA. (2009) 106:19940–5. 10.1073/pnas.0907898106 19918060 PMC2785271

[166] O'Brien KL Liu J King SL Sun YH Schmitz JE Lifton MA. Adenovirus-specific immunity after immunization with an Ad5 HIV-1 vaccine candidate in humans. Nat Med. (2009) 15:873–5. 10.1038/nm.1991 19620961 PMC2756115

[167] Duerr A Huang Y Buchbinder S Coombs RW Sanchez J del Rio C. Extended follow-up confirms early vaccine-enhanced risk of HIV acquisition and demonstrates waning effect over time among participants in a randomized trial of recombinant adenovirus HIV vaccine (Step Study). J Infect Dis. (2012) 206:258–66. 10.1093/infdis/jis342 22561365 PMC3490694

[168] Capone S Meola A Ercole BB Vitelli A Pezzanera M Ruggeri L. A novel adenovirus type 6 (Ad6)-based hepatitis C virus vector that overcomes preexisting anti-ad5 immunity and induces potent and broad cellular immune responses in rhesus macaques. J Virol. (2006) 80:1688–99. 10.1128/JVI.80.4.1688-1699.2006 16439526 PMC1367169

[169] Harro C Sun X Stek JE Leavitt RY Mehrotra DV Wang F. Safety and immunogenicity of the Merck adenovirus serotype 5 (MRKAd5) and MRKAd6 human immunodeficiency virus type 1 trigene vaccines alone and in combination in healthy adults. Clin Vaccine Immunol. (2009) 16:1285–92. 10.1128/CVI.00144-09 19605598 PMC2745015

[170] Green CA Sande CJ Scarselli E Capone S Vitelli A Nicosia A. Novel genetically-modified chimpanzee adenovirus and MVA-vectored respiratory syncytial virus vaccine safely boosts humoral and cellular immunity in healthy older adults. J Infect. (2019) 78:382–92. 10.1016/j.jinf.2019.02.003 30742894 PMC7172982

[171] Mensah VA Roetynck S Kanteh EK Bowyer G Ndaw A Oko F. Safety and immunogenicity of malaria vectored vaccines given with routine expanded program on immunization vaccines in Gambian infants and neonates: a randomized controlled trial. Front Immunol. (2017). 8:1551. 10.3389/fimmu.2017.01551 29213269 PMC5702785

[172] Bliss CM Drammeh A Bowyer G Sanou GS Jagne YJ Ouedraogo O Viral Vector malaria vaccines induce high-level T cell and antibody responses in West African children and infants. Mol Ther. (2017). 25:547–59. 10.1016/j.ymthe.2016.11.003 28153101 PMC5368405

[173] Bliss CM Bowyer G Anagnostou NA Havelock T Snudden CM Davies H. Assessment of novel vaccination regimens using viral vectored liver stage malaria vaccines encoding ME-TRAP. Sci Rep. (2018) 8:3390. 10.1038/s41598-018-21630-4 29467399 PMC5821890

[174] Afolabi MO Tiono AB Adetifa UJ Yaro JB Drammeh A Nebie I Safety and Immunogenicity of ChAd63 and MVA ME-TRAP in West African children and infants. Mol Ther. (2016). 24:1470–1477. 10.1038/mt.2016.83 27109630 PMC5010143

[175] Hodgson SH Ewer KJ Bliss CM Edwards NJ Rampling T Anagnostou NA. Evaluation of the efficacy of ChAd63-MVA vectored vaccines expressing circumsporozoite protein and ME-TRAP against controlled human malaria infection in malaria-naive individuals. J Infect Dis. (2015) 211:1076–86. 10.1093/infdis/jiu579 25336730 PMC4354983

[176] Ogwang C Kimani D Edwards NJ Roberts R Mwacharo J Bowyer G. Prime-boost vaccination with chimpanzee adenovirus and modified vaccinia Ankara encoding TRAP provides partial protection against plasmodium falciparum infection in Kenyan adults. Sci Transl Med. (2015) 7:286re285. 10.1126/scitranslmed.aaa2373 25947165 PMC4687051

[177] Tiono AB Nebie I Anagnostou N Coulibaly AS Bowyer G Lam E. First field efficacy trial of the ChAd63 MVA ME-TRAP vectored malaria vaccine candidate in 5-17 months old infants and children. PLoS ONE. (2018) 13:e0208328. 10.1371/journal.pone.0208328 30540808 PMC6291132

[178] Callendret B Vellinga J Wunderlich K Rodriguez A Steigerwald R Dirmeier U. A prophylactic multivalent vaccine against different filovirus species is immunogenic and provides protection from lethal infections with Ebolavirus and Marburgvirus species in non-human primates. PLoS ONE. (2018) 13:e0192312. 10.1371/journal.pone.0192312 29462200 PMC5819775

[179] Anywaine Z Whitworth H Kaleebu P Praygod G Shukarev G Manno D. Safety and Immunogenicity of a 2-dose heterologous vaccination regimen with Ad26.ZEBOV and MVA-BN-Filo Ebola vaccines: 12-month data from a phase 1 randomized clinical trial in Uganda and Tanzania. J Infect Dis. (2019) 220:46–56. 10.1093/infdis/jiz070 30796818 PMC6548900

[180] Milligan ID Gibani MM Sewell R Clutterbuck EA Campbell D Plested E. Safety and immunogenicity of novel adenovirus type 26- and modified vaccinia Ankara-vectored Ebola vaccines: a randomized clinical trial. JAMA. (2016) 315:1610–23. 10.1001/jama.2016.4218 27092831

[181] Salisch NC Izquierdo Gil A Czapska-Casey DN Vorthoren L Serroyen J Tolboom J. Adenovectors encoding RSV-F protein induce durable and mucosal immunity in macaques after two intramuscular administrations. NPJ Vaccines. (2019) 4:54. 10.1038/s41541-019-0150-4 31885877 PMC6925274

[182] Baden LR Walsh SR Seaman MS Tucker RP Krause KH Patel A. First-in-human evaluation of the safety and immunogenicity of a recombinant adenovirus serotype 26 HIV-1 Env vaccine (IPCAVD 001). J Infect Dis. (2013) 207:240–47. 10.1093/infdis/jis670 23125444 PMC3532831

[183] Barouch DH Liu J Peter L Abbink P Iampietro MJ Cheung A. Characterization of humoral and cellular immune responses elicited by a recombinant adenovirus serotype 26 HIV-1 Env vaccine in healthy adults (IPCAVD 001). J Infect Dis. (2013) 207:248–56. 10.1093/infdis/jis671 23125443 PMC3532832

[184] Barouch DH O'Brien KL Simmons NL King SL Abbink P Maxfield LF. Mosaic HIV-1 vaccines expand the breadth and depth of cellular immune responses in rhesus monkeys. Nat Med. (2010) 16:319–23. 10.1038/nm.2089 20173752 PMC2834868

